# The JAK-STAT pathway: from structural biology to cytokine engineering

**DOI:** 10.1038/s41392-024-01934-w

**Published:** 2024-08-21

**Authors:** You Lv, Jianxun Qi, Jeffrey J. Babon, Longxing Cao, Guohuang Fan, Jiajia Lang, Jin Zhang, Pengbing Mi, Bostjan Kobe, Faming Wang

**Affiliations:** 1 Center for Molecular Biosciences and Non-communicable Diseases Research, Xi’an University of Science and Technology, Xi’an, Shaanxi 710054 China; 2 Xi’an Amazinggene Co., Ltd, Xi’an, Shaanxi 710026 China; 3 CAS Key Laboratory of Pathogen Microbiology and Immunology, Institute of Microbiology, Chinese Academy of Sciences, Beijing, 100080 China; 4 The Walter and Eliza Hall Institute of Medical Research, Parkville, VIC 3052 Australia; 5 School of Life Sciences, Westlake University, Hangzhou, Zhejiang 310024 China; 6 Immunophage Biotech Co., Ltd, No. 10 Lv Zhou Huan Road, Shanghai, 201112 China; 7 School of Pharmaceutical Science, Hengyang Medical School, University of South China, Hengyang, Hunan 421001 China; 8 School of Chemistry and Molecular Biosciences, Institute for Molecular Bioscience and Australian Infectious Diseases Research Centre, University of Queensland, Brisbane, Queensland 4072 Australia

**Keywords:** Molecular biology, Structural biology

## Abstract

The Janus kinase-signal transducer and activator of transcription (JAK-STAT) pathway serves as a paradigm for signal transduction from the extracellular environment to the nucleus. It plays a pivotal role in physiological functions, such as hematopoiesis, immune balance, tissue homeostasis, and surveillance against tumors. Dysregulation of this pathway may lead to various disease conditions such as immune deficiencies, autoimmune diseases, hematologic disorders, and cancer. Due to its critical role in maintaining human health and involvement in disease, extensive studies have been conducted on this pathway, ranging from basic research to medical applications. Advances in the structural biology of this pathway have enabled us to gain insights into how the signaling cascade operates at the molecular level, laying the groundwork for therapeutic development targeting this pathway. Various strategies have been developed to restore its normal function, with promising therapeutic potential. Enhanced comprehension of these molecular mechanisms, combined with advances in protein engineering methodologies, has allowed us to engineer cytokines with tailored properties for targeted therapeutic applications, thereby enhancing their efficiency and safety. In this review, we outline the structural basis that governs key nodes in this pathway, offering a comprehensive overview of the signal transduction process. Furthermore, we explore recent advances in cytokine engineering for therapeutic development in this pathway.

## Introduction

**Fig. 1:**
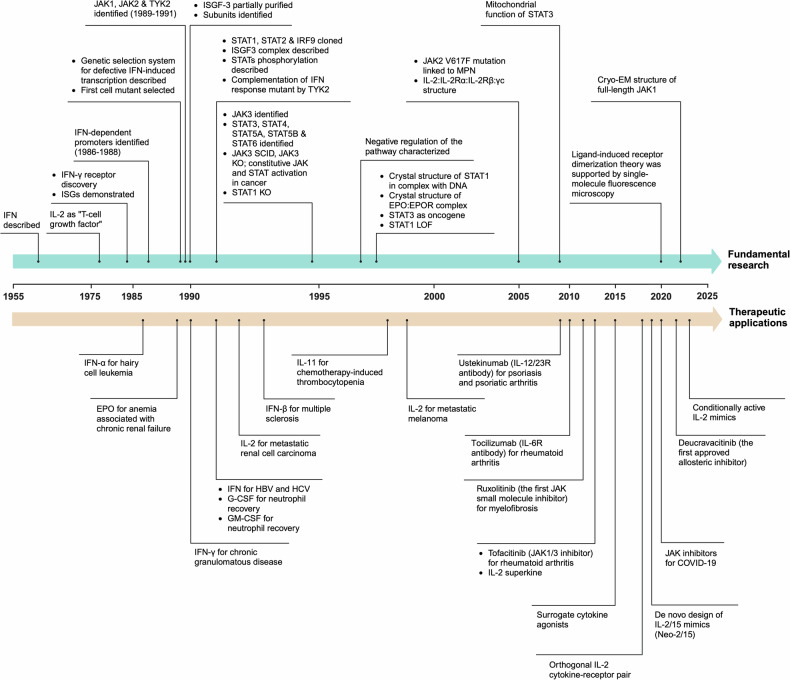
Timeline for milestones in the JAK-STAT signaling pathway: from fundamental research to therapeutic applications. Abbreviations: IL interleukin; ISG interferon-stimulated gene; TYK tyrosine kinase; ISGF interferon-stimulated gene factor; IRF interferon regulatory factor; SCID severe combined immunodeficiency; KO knockout; EPO erythropoietin; EPOR erythropoietin receptor; LOF loss of function; MPN myeloproliferative neoplasm; cryo-EM cryogenic electron microscopy; HBV hepatitis B virus; HCV hepatitis C virus; G-CSF granulocyte colony-stimulating factor; GM-CSF granulocyte-macrophage colony-stimulating factor; γc, common gamma chain

The story of the Janus kinase-signal transducer and activator of transcription (JAK-STAT) pathway can be traced back to the year 1957 when Alick Isaacs and Jean Lindenmann conducted investigations into how cells respond to interferons (IFNs).^1–3^ They discovered that influenza virus-infected chick embryo cells produced and released something in the surrounding fluid that induced resistance to infection in noninfected cells, which they named “interferon.” In the late 1980s, James E. Darnell’s group discovered an IFN-stimulation-dependent protein complex (later referred to as ISGF3, interferon-stimulated gene factor 3). This complex was first detected in the cytoplasm after 1 or 2 minutes of IFN-β treatment and then accumulated in the nucleus.^3–5^ In the early 1990s, Xinyuan Fu and colleagues purified ISGF3 and identified its constituent proteins, including a 48 kDa DNA-binding protein and three larger polypeptides (84, 91 and 113 kDa).^6^ Further invetigation by Xinyuan Fu revealed that ISGF3 contain conserved SH2 and SH3 domains and could be directly tyrosine phosphorylated both in vitro and in vivo in response to IFN-α. The tyrosine phosphorylation process could be inhibited by staurosporine and genistein, and phosphatase treatment of ISGF3 protein inhibited ISGF3 complex formation in vitro.^7^ ISGF3 was found to contain STAT1, STAT2, and IRF9 (interferon regulatory factor 9), thereby establishing the STAT protein family. These findings significantly advanced our understanding of the molecular mechanisms underlying IFN signaling, including the critical role of tyrosine phosphorylation in regulating the JAK-STAT pathway. Nearly simultaneously, JAK1, JAK2, and tyrosine kinase 2 (TYK2) were identified as non-receptor protein tyrosine kinases, but their functions were unknown.^8–11^ Laura Velazquez and colleagues demonstrated that molecularly complementing TYK2 can restore IFN response in a mutant IFN-α unresponsive cell line.^11^ Until then, these seminal studies linked cytokines, JAKs, and STATs. Over the next few decades, key components of this pathway were identified and functionally characterized, revealing a complete picture of this intricate puzzle (Fig. 1).^12–14^ The JAK-STAT pathway, despite its architectural simplicity—comprising cytokines, receptors, JAKs, and STATs—regulates a broad spectrum of biological functions such as cell proliferation, differentiation, migration, and apoptosis. Found ubiquitously in animals from the simple *Caenorhabditis elegans* to complex vertebrates, we now recognize the JAK-STAT pathway as a paradigm for transmitting information from the extracellular environment to the nucleus.^3,14^ The activation of this pathway is initiated by specific molecular interactions between cytokines and receptors, resulting in dimerization or multimerization of the receptors. This leads to the activation of JAKs, subsequent phosphorylation of STATs, and initiation of transcriptional events.^13,14^ However, when this pathway becomes dysregulated, it can have significant implications for cellular processes and contributes to the pathogenesis of diseases, such as immunodeficiency, inflammatory and autoimmune conditions, hematological disorders, and cancer. Given its broad effects on human biology and disease, the JAK-STAT pathway has emerged as an attractive target for the treatment of various disorders.^15–19^

Over the past three decades, significant progress has been made in advancing this pathway from basic research to medical applications (Fig. 1). One notable aspect of this advancement involves a fundamental understanding of the pathway through the lens of structural biology.^3,13,14^ Notably, the evolution of technologies like cryo-electron microscopy (cryo-EM) has propelled three-dimensional (3D) structural studies on proteins, including those integral to the JAK-STAT pathway. These technological breakthroughs have empowered us to visualize and scrutinize the intricate details of large protein complexes, whose structures are challenging to be solved using nuclear magnetic resonance (NMR) spectroscopy or crystallography, at near-atomic resolution, offering invaluable insights into the functional mechanisms of key signaling molecules.^20–24^ The elucidation of the structures of cytokines, receptors, JAKs and STATs provide insights into the molecular mechanisms governing cytokine-receptor recognition, pathway activation, and gene transcription.^24–26^ This enhanced understanding of the structure and molecular mechanisms within the JAK-STAT signaling has given rise to another promising field: cytokine engineering, which aims to tailor cytokines for a wide array of medical applications. In this review, we will summarize the structural foundation governing pivotal points in this pathway, striving to present a holistic view of the signal transduction process. Moreover, we will delve into a comprehensive discussion on the therapeutic frontiers with cytokine engineering.

### The JAK-STAT pathway as a drug target

The JAK-STAT pathway exhibits remarkable diversity and tissue-specific distributions, allowing it to perform various crucial roles in response to extracellular signaling proteins. It plays a fundamental part in the differentiation and maturation of diverse blood cell lineages, maintaining a balanced hematopoietic system with red blood cells, platelets, granulocytes, monocytes, and lymphocytes.^27–30^ Additionally, it regulates the development, activation, and function of immune cells, including B and T lymphocytes, natural killer (NK) cells, macrophages, and dendritic cells.^14^ Through modulating immune cell differentiation, activation, proliferation, and the production of immune mediators, the JAK-STAT pathway orchestrates effective immune responses, coordination of inflammation, immune homeostasis, surveillance against tumors, and elimination of aberrant cells.^14,15,31–34^ The importance of this pathway is highlighted by the fact that complete deficiencies in JAK1, JAK2, STAT3 or STAT5 result in lethality, while mutations in proteins within this pathway can lead to immune disorders, hematopoietic diseases, and cancer.^35–37^

Genetic variations in genes encoding cytokines, cytokine receptors, JAKs, and STATs have been identified as risk factors for immunodeficiencies and autoimmune disorders.^36–38^ Notably, mutations in JAK3 and tyrosine kinase 2 (TYK2) have been associated with primary immunodeficiency, particularly severe combined immune deficiency (SCID), characterized by impaired development or function of lymphocytes.^36,39,40^ The gene mutations in the common gamma chain (γc) are responsible for X-linked SCID, leading to diminished T cell and NK cell numbers and function, while B cell numbers are preserved but functionally impaired.^39–42^ Furthermore, mutations in STAT1 have been identified in patients with disseminated infections caused by Bacille Calmette–Guérin (BCG) or nontuberculous mycobacteria, while excessive STAT1 activation leads to exaggerated IFN-γ responses and inhibits interleukin-17 (IL-17) production, leading to the development of autoimmune conditions.^40,43^ Polymorphisms in STAT3 have been linked to Crohn’s disease (CD), psoriasis and Behçet’s disease.^44^ Similarly, polymorphisms in STAT4, activated by IL-12 and type I IFNs, have been linked to systemic lupus erythematosus (SLE), rheumatoid arthritis (RA), and Sjögren’s syndrome, and variants in STAT6 are associated with elevated IgE levels and atopic dermatitis (AD).^40,45^ In addition, certain JAK2 polymorphisms have been associated with Behçet’s disease, while single nucleotide polymorphisms in the TYK2 gene have been strongly linked to SLE, and also associated with other autoimmune conditions including CD, ulcerative colitis (UC), type 1 diabetes mellitus, multiple sclerosis (MS), and RA.^40,46,47^ The sustained activation of JAK1 and TYK2, resulting from increased serum IFNs and expression of IFN-inducible genes, is considered crucial in the molecular pathogenesis of SLE. Variants in the IL-12 and IL-23 signaling pathway have been linked to disorders such as CD and UC.^48,49^

JAK2 signaling is crucial for maintaining hematopoietic stem cell (HSC) homeostasis, as hematopoietic cytokines like erythropoietin (EPO), thrombopoietin (TPO) and granulocyte-macrophage colony-stimulating factor (GM-CSF) transmit their signals through JAK2. Conditional knockout of JAK2 leads to bone marrow failure, increased apoptosis, and loss of quiescence in HSC-enriched cells.^32,50,51^ On the other hand, gain-of-function (GOF) mutations in JAK2 are specifically associated with myeloproliferative neoplasms (MPNs). Examples of these neoplasms include polycythemia vera, essential thrombocythemia, and primary myelofibrosis (PMF). The most prevalent JAK2 mutation, V617F, is detected in more than 95% of polycythemia vera patients and in 32 to 57% of individuals with essential thrombocythemia or PMF.^32,50,52,53^ Apart from JAK mutations, the role of receptors linked to JAKs can be modified in cancer by chromosomal rearrangements or mutations, resulting in continuous JAK activation. For instance, myelofibrosis is also associated with activating mutations in the thrombopoietin receptor, while B-cell acute lymphoblastic leukemia (ALL) can involve chromosomal rearrangements affecting the cytokine receptor. Furthermore, dysregulation of specific STATs also plays a significant role in leukemic hematopoiesis. Among these STATs, STAT1 has been identified as a promoter of leukemia development, while STAT5 plays a crucial role in the progression of malignancies affecting myeloid and lymphoid lineages. Constitutive activation of STAT1, STAT3, and STAT5 has been observed in cells derived from acute leukemias. Mutations in STAT5A leading to its constitutive activation and formation of stable tetramers have been associated with multilineage leukemias.^54,55^

Apart from hematopoietic cancers, the JAK-STAT signaling pathway is widely involved in multiple types of solid tumors.^35,40^ Extensive research has focused on investigating the potential involvement of the pathway in hepatocellular carcinoma (HCC) pathogenesis. Inflammatory hepatocellular adenomas (IHCAs) are benign liver tumors defined by the presence of inflammatory infiltrates and elevated expression of inflammatory proteins in tumor hepatocytes.^56,57^ Sixty percent of IHCAs are characterized by GOF mutations on glycoprotein 130 (gp130), resulting in the activation of IL-6 signaling in the absence of ligands, while 12% of IHCAs are attributed to GOF mutations on STAT3.^58,59^ Constitutive activation of STAT3 has been observed in a substantial proportion of HCC cases, establishing it as an oncogene that promotes HCC development.^35,60,61^ The transcriptional activity of STAT3 leads to the upregulation of genes associated with different cancer hallmarks. Notably, cells with constitutively activated STAT3 display heightened levels of cyclin D1, a protein that drives cell cycle progression from G1 to S phase, contributing to uncontrolled cell proliferation in HCC.^35,62^ Moreover, STAT3 exerts pro-angiogenic effects in HCC by regulating the expression of various pro-angiogenic factors within the tumor microenvironment (TME). Elevated IL-6 levels and aberrant STAT3 activation have further been observed in head and neck cancers and gastric cancers, contributing to increased tumor cell proliferation, survival, metastasis, and immunosuppression.^63–65^ In pancreatic cancer, hyperactive STAT3 is associated with increased signaling induced by IL-22 and suppression of suppressor of cytokine signaling (SOCS) 3, resulting in enhanced invasion, migration, and angiogenesis.^66^ Recent evidence underscores the JAK-STAT pathway’s crucial role in promoting lineage plasticity, enabling cancers to resist targeted therapies.^67,68^ For instance, several studies have demonstrated that the JAK-STAT signaling significantly contributes to the lineage transition observed in prostate cancer, leading to a shift from adenocarcinoma to neuroendocrine cancer.^68–71^

Due to its critical role in human health and disease, therapeutic development targeting the JAK-STAT pathway has received considerable attention. Various strategies have been explored to modulate the pathway and restore its normal function.^49,72^ One major approach involves the development of small molecules inhibitors that target the JAK-STAT pathway. These inhibitors have been widely utilized in the treatment of autoimmune diseases such as RA, CD, and UC, as well as hematopoietic diseases like PMF.^73–76^ Ruxolitinib (Jakafi; Incyte/Novartis), the first small molecule JAK inhibitor, was FDA-approved in November 2011 for treating patients with intermediate or high-risk myelofibrosis, including PMF, post-polycythemia vera myelofibrosis, and post-essential thrombocythemia myelofibrosis.^77–80^ A year later, the JAK inhibitor tofacitinib, developed by Pfizer, received approval for the treatment of RA, demonstrating a consistent safety profile in long-term follow-up studies, either as monotherapy or in combination therapy.^81,82^ Over the following years, the indications for tofacitinib expanded significantly, with FDA approvals for treating UC, psoriatic arthritis (PsA), and alopecia areata (AA), as well as ongoing clinical trials for psoriasis, graft-versus-host disease (GVHD), CD, and SLE.^83–87^ Following the success of ruxolitinib and tofacitinib, the development of small molecule drugs targeting the JAK-STAT pathway became a focal point, with numerous potent inhibitors exhibiting excellent pharmacological properties.^17,44,49,88–92^ Currently, the FDA has approved over 10 small molecules targeting this pathway for a range of conditions, with over 40 inhibitors at various stages of clinical development.^18,93–100^ Many of these inhibitors demonstrate exceptional selectivity for specific JAK proteins, leading to improved safety profiles.^101–106^ For instance, upadacitinib, developed by AbbVie, is a JAK1 selective inhibitor (JAK1/JAK2/JAK3/TYK2 IC_50_ = 43/200/2300/4700 nM) approved for treating autoimmune diseases such as RA, AD, and PsA, showing a remarkable safety profile.^98,107^ Fedratinib, a potent JAK2 selective inhibitor developed by Bristol Myers Squibb, with an IC50 of 3 nM for JAK2 and 35 ~ 334 nM for other JAKs, was FDA-approved for myelofibrosis treatment in 2019.^95,104,108^ Another significant small molecule inhibitor targeting the JAK-STAT pathway is deucravacitinib, the first allosteric inhibitor targeting the pseudokinase (PK) domain of TYK2, approved by the FDA for psoriasis treatment.^109–111^ Following the success of deucravacitinib, several other allosteric inhibitors have been developed, showing promising results in treating diseases related to the JAK-STAT pathway.^88,112–116^

In 2019, the emergence of the severe acute respiratory syndrome coronavirus 2 (SARS-CoV-2) pandemic spread widely across the globe, presenting a significant threat to human life and health.^117–120^ Following SARS-CoV-2 infection, many individuals developed cytokine storm and cytokine release syndrome, which are life-threatening systemic inflammatory conditions characterized by rapid elevation of circulating cytokine levels and hyperactivation of immune cells. Very high levels of IL-2, IL-7, IL-10, tumor necrosis factor (TNF)-α, and granulocyte colony-stimulating factor (G-CSF) were observed in coronavirus disease 2019 (COVID-19) patients requiring intensive care.^121^ These syndromes are major contributors to the severity and mortality of COVID-19.^122–125^ Most of these cytokines signal through the JAK-STAT pathway. Therefore, blocking this pathway has been proposed as a strategy to manage these hyperinflammatory conditions. Small molecule JAK inhibitors, which attenuate the activity of this pathway, have demonstrated notable efficacy in alleviating symptoms and reducing mortality rates among COVID-19 patients experiencing cytokine storm and cytokine release syndrome.^126–131^ Positive results from clinical trials led to the FDA recommendation of baricitinib in certain settings of COVID-19 and its recommendation in the U.S. NIH practice guideline for the treatment of COVID-19.^132^ Similar conclusions were reached by the expert committee of the World Health Organization (WHO), recommending baricitinib in severe and critical COVID-19 since the eighth version of its ‘A living WHO guideline on drugs for covid-19’ from January 2022.^133^ In addition, tofacitinib also received recommendation by the U.S. NIH in case of baricitinib unavailability.^134^

In addition to JAK inhibition, small molecules that competitively bind to the phosphorylated tyrosine-binding site on the Src homology 2 (SH2) region of STATs can hinder the recruitment and phosphorylation of STATs or prevent their conformational changes necessary for functioning as transcription factors. This interference leads to the dampening of downstream signaling, presenting a novel strategy for targeting the JAK-STAT pathway. Several small molecules have been developed to effectively bind to STATs, with some designed to specifically target STAT3, STAT5, or STAT6. These compounds have shown promise in animal models for treating diseases associated with the JAK-STAT pathway, such as allergic airway conditions and breast cancer.^134–140^

Another notable therapeutic strategy involves the development of agonists or antagonists that directly interact with the extracellular components of the JAK-STAT signaling cascade.^30,87,141–145^ These agonists or antagonists primarily consist of macromolecules, such as natural cytokines, engineered cytokines, cytokine mimics, or antibodies; they promote or inhibit specific downstream signaling events, thereby influencing cellular responses.^146,147^

Inflammation is a crucial defense mechanism against harmful irritants, playing a vital role in eliminating injury factors and promoting tissue regeneration. However, when inflammation becomes persistent and excessive, it can contribute to the development of autoimmune diseases. Cytokines, which are key mediators of immune responses, play a significant role in autoimmunity.^148,149^ Neutralizing inflammatory cytokines or blocking their receptor function has proven to be an effective therapeutic strategy for autoimmune diseases.^30^ For example, in autoimmune conditions like psoriasis, IL-6 and transforming growth factor β (TGFβ) stimulate the differentiation of naive CD4^+^ T cells into IL-17-producing T cells.^30,150^ IL-6 is also implicated in other autoimmune diseases, including experimental autoimmune encephalomyelitis (EAE), collagen-induced arthritis (CIA), RA, inflammatory bowel disease (IBD), and psoriasis.^151,152^ Antibodies targeting IL-6 or its receptor, such as Tocilizumab, Siltuximab, Sirukumab, Olokizumab, and Sarilumab, have been approved by the FDA or are undergoing clinical trials for various autoimmune conditions, including RA, CD, ankylosing spondylitis, and Castleman’s disease.^30,141,152–158^ IL-23, activated by TNF, plays a crucial role in amplifying the production of inflammatory cytokines and the generation of pathogenic T helper 17 (Th17) cells, contributing to diseases like CD, RA, psoriasis, and other inflammatory conditions.^142,159^ Antibodies that bind to IL-23, including those targeting p40 (Ustekinumab and Briakinumab) or p19 (Tildrakizumab, Guselkumab, Risankizumab, Mirikizumab, LY2525623 and AMG139), have been developed for the treatment of autoimmune disorders like psoriasis, psoriatic arthritis, and CD.^30,87,142,143,160–163^ Type 1 IFNs play a critical role in the pathogenesis of autoimmune diseases, particularly SLE. Clinical evidence from SLE patients has shown a significant increase in the expression of type 1 IFN signatures.^164^ Recently, Anifrolumab, a human monoclonal antibody that targets IFNAR1 to inhibit the biological function of all type 1 IFNs, has received approval for the treatment of moderate to severe SLE.^165^ Clinical trials have consistently demonstrated that Anifrolumab provides various clinical benefits, including reduced disease activity, decreased usage of oral corticosteroids, and fewer annual flares.^144,166,167^

The interest in therapeutic applications of cytokines surged after the 1980s with the identification of more cytokines and advancements in cytokine biology, coupled with the development of recombinant protein techniques.^147,168^ So far, several cytokines within this pathway have obtained FDA approval for a range of therapeutic purposes, including the treatment of hematopoietic disorders, antiviral interventions, cancer therapy, and other applications.^145,169–173^ For example, hematopoietic cytokines such as EPO, G-CSF, GM-CSF, and IL-11 play vital roles in regulating the production, survival, proliferation, and differentiation of hematopoietic cells.^174–177^ The regulation of hematopoiesis by these cytokines is tightly controlled and finely balanced to ensure the production of an appropriate number and type of hematopoietic cells in response to physiological demands. Dysregulation or deficiencies in these cytokines can lead to hematopoietic disorders, such as anemia, neutropenia, or thrombocytopenia.^178,179^ These cytokines have received approval for medical use, and their clinical application has played a crucial role in effectively managing diverse hematopoietic conditions including. For example, recombinant EPO products, such as epoetin alfa and darbepoetin alfa, have obtained FDA approval and find extensive use in different clinical settings for the management of anemia-associated conditions.^180–182^ Recombinant GM-CSF has been primarily licensed for myeloid reconstitution after autologous or allogeneic blood and bone marrow transplantation. Additionally, it is employed to expedite neutrophil recovery following chemotherapy for acute myeloid leukemia and as a medical countermeasure for treating individuals exposed to high levels of radiation resulting in severe myelosuppression.^183,184^ The IFN family of cytokines serves as the body’s first line of defense against viral infections. Infected cells produce and release these cytokines, stimulating innate antimicrobial responses in nearby cells and activating interferon-stimulated genes that promote antiviral activity.^185,186^ Consequently, they have emerged as promising candidates for antiviral therapeutics including hepatitis B virus (HBV) and hepatitis C virus (HCV).^185–190^

Cytokines, as crucial immune regulators, have intricate connections with tumorigenesis. Many cytokines function by modulating the immune system to eliminate aberrant cells, thereby preventing the occurrence of cancer.^40,191–193^ One significant cytokine in cancer therapy is IL-2, which regulates and activates various immune cells, including T, B, NK, and dendritic cells.^194^ High-dose IL-2 has been approved by the FDA for the treatment of metastatic kidney cancer in 1992 and metastatic melanoma in 1998.^195,196^ Its therapeutic efficacy is attributed to its ability to stimulate the immune system and enhance the activity of immune cells. By promoting the expansion and activation of cytotoxic T cells and NK cells, IL-2 facilitates the recognition and elimination of cancer cells. This significant effect of IL-2 serves as landmark clinical evidence, demonstrating the effectiveness of purely immunological manipulation in driving tumor eradication in humans.^193,196,197^ In the last several decades, advances in tumor biology have unveiled the complex relationship between the immune system, healthy cells, and malignant cells. This understanding has paved the way for the concept of immunosurveillance, underscoring the immune system’s remarkable ability to recognize and eliminate transformed cells. Cytokines, as crucial mediators, play a pivotal role in facilitating interactions between immune and non-immune cells within TME. These interactions intricately shape the dynamics of tumor development, progression, and immune responses.^40,193,198,199^

### Cytokine binding and receptor activation

**Fig. 2:**
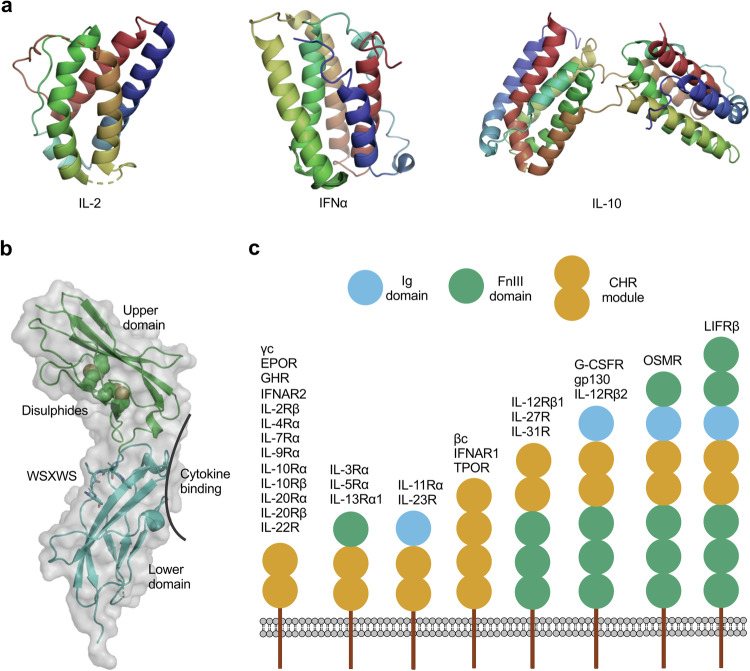
The JAK-STAT pathway cytokines and their receptors. **a** Exemplary structures of typical cytokines, colored as a spectrum from the N- (blue) to the C-terminus (red). **b** The structure of the cytokine-binding homology region (CHR). **c** Domain composition of common cytokine receptors

Over 50 cytokines have been identified as ligands for the JAK-STAT pathway, including interleukins, interferons (IFNs), colony-stimulating factors (CSFs), EPO, TPO and growth hormone (GH). These extracellular signaling molecules bind to two major classes of receptors on the cell surface, initiating the activation of the JAK-STAT signaling cascade (Table 1). Therefore, they can be classified into two classes based on the type of receptor they engage: class I cytokines such as EPO, TPO, IL-2, IL-3 and IL-6 family cytokines; and class II cytokines such as IFNs and IL-10 family cytokines (Table 1).^25,200,201^ Class I cytokines typically consist of four helices connected by long loops, oriented into a unique up-up-down-down topology that is only found in the helical cytokines; class II cytokines have these loops replaced by additional helices, resulting in 5–6 helices (Fig. 2a). While the majority of these cytokines typically range from 100 to 200 amino acids in length and exist as monomers, there are a few exceptions such as IL-10, IL-26, and IFNγ, which present as dimers, with each monomer having a length similar to conventional monomeric cytokine.^13,25,202,203^

**Table 1:** Classification of cytokines, their receptor engagement, downstream JAKs and STATs, and main functions

Cytokines	Receptors	JAKs	STATs	Major functions
**Type I, homodimeric cytokines**				
EPO	EPOR:EPOR	JAK2	STAT5	Control of erythropoiesis
GH	GHR:GHR	JAK2	STAT3 STAT5	Stimulation of cell division of chondrocytes and IGF-1
G-CSF	G-CSFR:G-CSFR	JAK1/JAK2/TYK2	STAT3	Stimulates granulocyte production, mobilizes stem cells
LEP	LEPR:LEPR	JAK2	STAT3 STAT5	Regulates appetite and energy expenditure
PRL	PRLR:PRLR	JAK2	STAT5	Metabolism and in modulation of the immune response, milk production
TPO	TPOR:TPOR	JAK2	STAT1 STAT3 STAT5	Regulation of the differentiation of megakaryocytes and platelets
**Type I, βc cytokines**				
IL-3	IL-3Rα:βc	JAK2	STAT3 STAT5 STAT6	Differentiation of multipotent hematopoietic stem cells, proliferation of all cells in the myeloid lineage
IL-5	IL-5Rα:βc	JAK2	STAT3 STAT5 STAT6	B cell development, eosinophils
GM-CSF	GM-CSFRα:βc	JAK2	STAT3 STAT5	Growth of macrophages and granulocytes, enhances T cell and dendritic-cell function, stimulation and differentiation of stem cells
**Type I, γc cytokines**				
IL-2	IL-2Rα:IL-2Rβ:γc IL-2Rβ:γc	JAK1/JAK3	STAT3 STAT5	Immune response, T cell differentiation
IL-4	IL-4Rα:γc IL-4Rα:IL-13Rα1	JAK1/JAK3/TYK2	STAT5 STAT6	Th2 differentiation
IL-7	IL-7Rα:γc	JAK1/JAK3	STAT3 STAT5	T, B cells development and homeostasis
IL-9	IL-9Rα:γc	JAK1/JAK3	STAT1 STAT3 STAT5	Stimulates T, B, NK cells
IL-13	IL-4Rα:IL-13Rα1	JAK1/TYK2	STAT6	Airway epithelia, allergic response
IL-15	IL-15Rα:IL-2Rβ:γc	JAK1/JAK3	STAT3 STAT5	Induction of memory T, NK cells proliferation
IL-21	IL-21Rα:γc	JAK1/JAK3	STAT1 STAT3 STAT5	Stimulates T, B, NK cells
TSLP	IL-7Rα:TSLPR	JAK1/JAK3	STAT5	Inflammatory, stimulates T, B cells
**Type I, gp130 cytokines**				
IL-6	IL-6Rα:gp130	JAK1/JAK2/TYK2	STAT1 STAT3	Pleiotropic, hematopoiesis, acute phase response, lymphoid differentiation
IL-11	IL-11Rα:gp130	JAK1/JAK2/TYK2	STAT3	Hematopoiesis, bone remodeling
IL-27	IL-27Rα:gp130	JAK1/JAK2/TYK2	STAT1 STAT3	Enhance Th1 cell responses and inhibit Th17 cell responses
IL-31	IL-31Rα:OSMR	JAK1/JAK2/TYK2	STAT3	Inflammatory, cell-mediated immunity
CNTF	LIFRβ:CNTFR:gp130	JAK1	STAT3	Neuronal growth factor
CT-1	LIFRβ:CNTFR:gp130	JAK1	STAT3	Cardiac myocytes growth factor
LIF	LIFRβ:gp130	JAK1/JAK2/TYK2	STAT3	Pleiotropic, blastocyst implantation, bone remodeling, central nervous system
OSM	OSMR:gp130	JAK1/JAK2/TYK2	STAT3	Pleiotropic, bone formation
**Type I, IL-12/23**				
IL-12p35	IL-12Rβ1:IL-12Rβ2:p40	JAK2/TYK2	STAT4	Differentiation of naive T cells into Th1 cells, stimulates T, NK cells
IL-23p19	IL-12Rβ1:IL-23R:p40	JAK2/TYK2	STAT3 STAT4	Inflammation
**Type II, IFN family cytokines**				
IFNα1, IFNα2 IFNα4, IFNα5, IFNα6, IFNα7, IFNα8, IFNα10, IFNα13, IFNα14, IFNα16, IFNα17, IFNα21, IFNβ, IFNε, IFNκ, IFNω	IFNAR1:IFNAR2	JAK1/TYK2	STAT1 STAT2	Antiviral, antiproliferative, and antitumor activities, enhance immunity against infections
IFNγ	IFNGR1:IFNGR2	JAK1/JAK2	STAT1	Pro-inflammatory, enhance immunity against infections and drive autoimmunity
IFNλ1, IFNλ2, IFNλ3	IL-10Rβ:IL-28R	JAK1/TYK2	STAT1 STAT2	Anti-viral, similar to type I but acts on fewer cell-types
**Type II, IL-10 family cytokines**				
IL-10	IL-10Rα:IL-10Rβ	JAK1/TYK2	STAT3	Anti-inflammatory, inhibits macrophage activation
IL-19	IL-20Rα:IL-20Rβ	JAK1/TYK2	STAT3	B cell activation
IL-20	IL-20Rα:IL-20Rβ IL-20Rβ:IL-22R	JAK1/TYK2	STAT3	wound healing, proliferation of epithelial cells
IL-22	IL-10Rβ:IL-22R	JAK1/TYK2	STAT3	Promote endothelial barrier immunity, augment IL-17 function
IL-24	IL-20Rα:IL-20Rβ IL-20Rβ:IL-22R	JAK1/TYK2	STAT3	Inflammatory
IL-26	IL-10Rβ:IL20Rα	JAK1/TYK2	STAT3	Antimicrobial, Th17 cytokine

The class I receptors, also known as hematopoietin receptors, constitute the largest group within the cytokine receptor family, with over 30 members encoded in the human genome (Table 1). A wide variety of interleukins, hematopoietins, and growth factors are known to bind to these receptors.^25,204,205^ Within this receptor group, there are two main types: shared receptors and cytokine-specific receptors. The class I cytokine receptor family comprises three major shared receptors: the common beta chain (βc), the γc, and gp130 (Table 1). These shared receptors participate in forming cytokine-receptor complexes for nearly 20 different cytokines.^25^ Although shared receptors alone generally exhibit low or no binding affinity to cytokines, they function in conjunction with cytokine-specific receptors, which have high affinities for specific cytokines. By contrast, certain cytokine-specific receptors can independently form functional cytokine-receptor complexes.^13,25^ The class II receptor family, similar to class I receptors, consists of both shared chains and cytokine-specific chains (Table 1). However, class II receptors exhibit a lower degree of specificity compared to class I receptors. Multiple cytokines can bind to a single receptor pair, and conversely, a single cytokine can bind to multiple receptor pairs in certain cases.^13,204–206^ For example, type I IFNs including IFNα, IFNβ, IFNε, IFNκ and IFNω all bind to the IFNAR1:IFNAR2 heterodimer, while IL-20 and IL-24 can bind to both the IL-20Rβ:IL-20Rα heterodimer and the IL-20Rβ:IL-22R heterodimer (Table 1).^13^ Another type of receptors are decoy receptors, which can arise through various mechanisms, including ectodomain shedding, where proteases cleave the extracellular domain of membrane-bound proteins to create decoy receptors, alternative splicing that generates diverse protein isoforms, and viral proteins mimicking host decoy receptors to interfere with the host immune response, acting as natural antagonists of the cell.^207–211^ Decoy receptors bind to cytokines and prevent them from forming functional cytokine receptor complexes, thereby inhibiting their signaling activities. These decoy receptors play a crucial role in regulating cytokine-mediated responses and maintaining immune balance.^211,212^

Cytokine receptors are transmembrane proteins with an extracellular N-terminal domain and an intracellular C-terminal domain, adopting a type I membrane topology. The extracellular domain plays a crucial role in cytokine recognition, while the intracellular domain contains a long tail that facilitates the binding of JAKs and recruitment of STATs.^25,213^ However, certain receptors, such as IL-2Rα, IL-6Rα, IL-11Rα and IL-15Rα, lack the necessary regions for direct binding of JAKs or STATs.^13,25,214–216^ These receptors are primarily responsible for ligand binding and the formation of cytokine-receptor complexes, relying on their partner receptors to transmit information and activate downstream signals. A common feature of the extracellular segments of cytokine receptors is their modular architecture, which includes one or more cytokine-binding homology regions (CHRs) spanning approximately 200 residues.^13,25,203^ However, a few exceptions like IL-2Rα and IL-15Rα possess one or two Sushi domains instead.^215,216^ The CHR module consists of two consecutive fibronectin type-III (FnIII) domains, small domains of approximately 100 residues, arranged in a β-sandwich structure comprising three-stranded and four-stranded β-sheets, connected by a linker region. Within the upper domain of the CHR module, there are four conserved cysteine residues, forming interstrand disulfide bonds (Fig. 2b). The lower domain is characterized by a conserved ‘W-S-X-W-S’ motif, present exclusively in the CHR modules of class I cytokine receptors (Fig. 2b). Although these amino acids do not directly participate in cytokine interaction, they play a vital role in maintaining the overall tertiary structure of the receptors.^13,25,204,213^ The binding site for cytokines is situated at the junction between the two FnIII domains within the CHR module. Flexible and variable loops originating from each domain contribute to the specificity of cytokine binding at this site. Despite some common features, cytokine receptors exhibit significant structural diversity (Fig. 2c).^13,25^ For example, the extracellular domains of cytokine receptors can vary in their composition and organization. Some receptors, like EPOR, GHR, and γc, have a relatively simple architecture with a single CHR unit.^13,25,213,217^ By contrast, receptors like leptin receptor (LEPR), IL-12Rβ2, IL-23R, leukemia inhibitory factor receptor (LIFR), and Oncostain M receptor (OSMR) are the largest cytokine receptors, comprising multiple domains.^13,25,218–220^ For instance, OSMR follows an FnIII-Ig(immunoglobulin)-CHR-FnIII-FnIII-FnIII architecture, while LIFR features a FnIII-FnIII-Ig-CHR-FnIII-FnIII-FnIII architecture.

Despite receptors in the JAK-STAT pathway displaying significant diversity in their domain compositions, their binding models can be categorized into a few patterns, based on their binding stoichiometries. The simplest model for cytokine-receptor binding involves one cytokine binding to a receptor homodimer, as observed for EPO, TPO, and GH complexes.^213,221–224^ In this case, two different sites on the cytokine can interact with the same receptor, resulting in a cytokine:receptor 1:2 complex. Unlike homodimeric receptors, the prevailing model for cytokine receptor binding involves the interaction between a cytokine and a receptor heterodimer. In this context, most cytokines initially bind to their specific receptors with high affinity and subsequently associate with common chain receptors, albeit with lower affinity.^13,25^ Here we present the binding models for JAK-STAT pathway cytokines through specific examples.

**Fig. 3:**
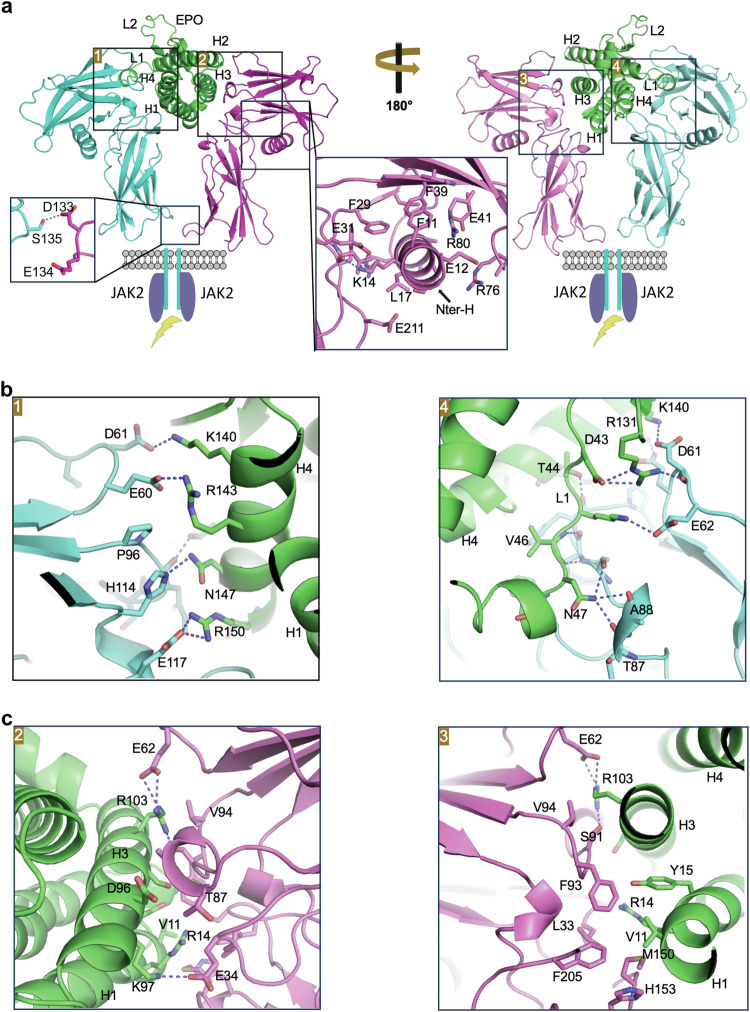
Crystal Structure of the EPO:EPOR complex (PDB ID: 1EER). **a** Overall Structure of the EPO:EPOR complex from both “front” (left) and “back” views (right). The interfaces between the cytokine and receptor are boxed. H1, H2, H3, and H4 correspond to helices 1, 2, 3, and 4 of EPO, while L1 and L2 designate loop 1 and loop 2 of EPO, respectively. The N-terminal helix (Nter-H) of EPOR plays a crucial role in maintaining receptor stability, forming numerous contacts with both the upper and lower domains of the receptor. The lower domains of EPORs are in close proximity, and a hydrogen bond forms between S135 of the first EPOR and D133 of the second EPOR. **b** Cytokine-receptor interaction details for site 1. **c** Cytokine-receptor interaction details for site 2

EPO is a vital hematopoietic cytokine in humans, playing a crucial role in orchestrating the differentiation and proliferation of precursor cells, ultimately contributing to the formation of red blood cells.^225^ The EPO:EPOR interaction represents the simplest model for cytokine receptor binding. Despite its asymmetrical structure, EPO can engage with two identical EPORs. EPO binds to the first EPOR at site 1 with high affinity (K_D_ ≈ 1 nM) and to the second EPOR at site 2 with lower affinity (K_D_ ≈ 1 μM).^226^ The crystal structure of the EPO:EPOR complex, was solved by Rashid Syed and colleagues, reveals that the extracellular domain of EPOR comprises a CHR module primarily containing β strands, with the exception of an observed helix at the N-terminus (Fig. 3a).^227^ This helix appears critical for maintaining the EPOR structure. It situates at the juncture between two FnIII domains, a flexible region where it interacts with both FnIII domains to enhance stability of the receptor (Fig. 3a). EPO is held by two EPOR molecules through contact at two sites: site 1, composed of EPO residues from helices 1 and 4, along with parts of loops 1 and 2 (Fig. 3b); and site 2, where residues are located in helices 1 and 3 (Fig. 3c). Cytokine-receptor interactions at both sites involve a series of hydrophobic and charged interactions. Notably, more protein contacts are observed between site 1 and EPOR, potentially explaining the higher affinity of site 1 for EPOR (Fig. 3). This binding of EPO to EPORs results in the close proximity of the lower domain of the CHR, with a distance of less than 10 Å. A hydrogen bond is observed between the side chain of S135 from the first EPOR and D133 from the second EPOR (Fig. 3a). This intimate proximity between the lower domains of EPOR appears to be important for stabilizing the EPO-receptor complex.

**Fig. 4:**
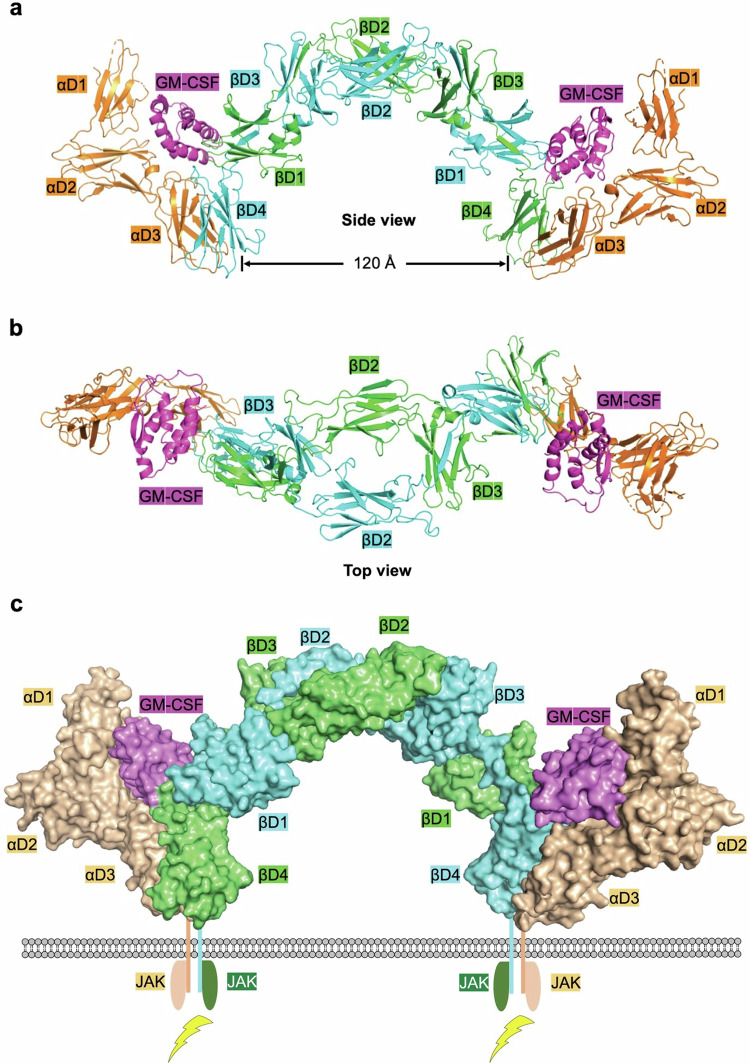
Structure of the cytokine-receptor complex of βc cytokines exemplified by the GM-CSF-receptor complex (PDB ID: 4NKQ). Domain 1 (D1, orange) of GM-CSFRα is modeled using D1 of GM-CSFRα from the GM-CSF:GM-CSFRα complex (PDB ID: 4RS1). **a** Hexamer of the GM-CSF-receptor complex from a side view. The CHR modules of GM-CSFRα (D2 and D3, orange) interact with GM-CSF (colored in magenta) at site 1, while the CHR modules of βc (D4 from itself and D1 from the partner βc) interact with GM-CSF at site 2. Each βc dimer is bound by two molecules of JAK2, but two JAK2 molecules are distant from each other, as βc D4 domains are approximately 120 Å apart. **b** Cytokine-receptor hexamer of the GM-CSF-receptor complex from a top view. The CHR modules of βc are bridged by the D2 and D3 domains of βc. **c** Signaling complex for βc cytokines exemplified by the GM-CSF-receptor complex. The intracellular domains of both GM-CSFRα and βc associate with JAKs, which is critical for signaling

βc is a shared cytokine receptor for IL-3, IL-5 and GM-CSF. The βc family cytokines modulate inflammatory responses, aiding in the effective clearance of pathogens while also contributing to the pathology associated with chronic inflammation. They exert control over the growth, differentiation, migration, and effector functions of various hematopoietic cells in the bone marrow, blood, and inflammatory sites. Dysregulated signaling, characterized by excessive or aberrant activity, carries the potential to initiate chronic inflammatory conditions and myeloid leukemias.^228–231^ The extracellular domain structure of βc was first resolved without cytokine binding, revealing a dimeric arrangement.^232,233^ Subsequently, in 2008, the structure of GM-CSF in complex with GM-CSFRα and βc was determined, with further improvements to the model’s quality in 2016, indicating the formation of a hexamer consisting of two copies each of GM-CSF, GM-CSFRα, and βc molecules.^234,235^ The cytokine-receptor complex structure reveals that βc exhibits an interlocked dimeric structure with 2-fold symmetry in an arch-bridge shape (Fig. 4a).^236^ Each copy of βc molecule consists of four domains (D1 to D4) in a FnIII fold. Among these domains, D2 and D4 can fold independently, while D1 and D3 of βc each require a β-sheet from the partner βc molecule to form a complete FnIII domain (Fig. 4a and b). The CHR module of βc, crucial for cytokine binding, is formed by D4 from itself and D1 from its partner molecule. The D2 and D3 domains of the dimeric βc are not directly involved in cytokine binding; rather, they serve as connectors, linking two assembled CHR modules at both ends.^234,235^ The extracellular segment of GM-CSFRα comprises an Ig domain at the N-terminus followed by a CHR module (D2 and D3), both involve in cytokine binding. D3 of GM-CSFRα makes direct contact with D4 of βc, potentially enhancing the stability of the GM-CSF-receptor hexamer.

For two decades, it was wrongly assumed that the specific receptors of βc family cytokines (IL-3Rα, IL-5Rα, and GM-CSFRα) did not link with JAKs and that signaling of these cytokines depended only on the shared βc.234-236 However, in the dimeric βc structure, the intracellular domains are approximately 120 Å apart, which is too far to enable signaling (Fig. 4a). Higher-order oligomeric states (dodecamer) were identified in the crystal lattice, potentially bringing the two βc intracellular domains closer together. Therefore, the dodecamer was proposed as the smallest unit responsible for GM-CSF signaling.^234–237^ This incorrect notion persisted until Christopher Garcia's group published a paper on the organizing principle of common beta cytokine signaling.^238^ The authors used cryo-EM to establish the structures of the βc family cytokine-receptor complex at 3.6–3.8 Å resolutions.^238^ The cytokine-receptor complex is a hexameric structure with two copies of the cytokine, receptor, and βc molecules, comparable to the crystal structure. Cryo-EM did not reveal increased oligomeric states, which were thought to bring the two βc intracellular domains closer together for signaling. Thus, the dodecameric cytokine-receptor structure is most likely due to crystal constraints and does not present in physiological settings. This conclusion was reinforced by single-molecule imaging experiments.^238^ Live-cell micropatterning tests were used to determine if the specific receptors could connect with JAKs, and the results revealed that all three unique receptors could bind JAKs significantly above baseline. The role of particular receptor intracellular regions in signaling was verified by connecting the transmembrane region and intracellular domains of βc family cytokine receptors to the extracellular domain of the orthogonal IL-2 receptor system.^238^ When orthogonal IL-2 was added, dose-dependent signaling occurred. The βc family cytokine-receptor complex, with its hexameric shape, is sufficient for cytokine signaling (Fig. 4c), indicating that particular receptors interact with JAKs and contribute to signaling.

**Fig. 5:**
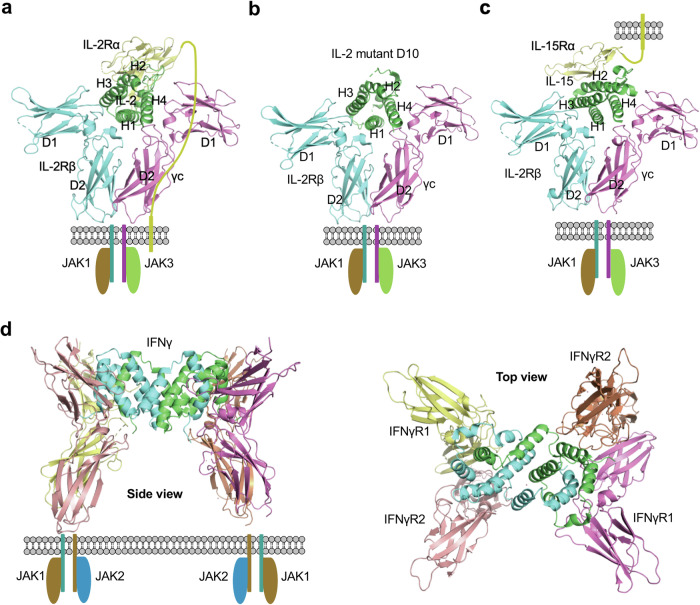
Cytokine-receptor complex structurers of γc cytokines and dimeric cytokines. Upper domains (domain 1) of receptors, are labeled D1, while lower domains (domain 2) are labeled D2. Helices 1, 2, 3, and 4 are labeled as H1, H2, H3, and H4, respectively. **a** The structure of IL-2 in complex with IL-2Rα, IL-2Rβ, and γc (high-affinity form, PDB ID: 2B5I). **b** The structure of IL-2 mutant D10 in complex with IL-2Rβ, and γc (PDB ID: 3QAZ). **c** Structure of the IL-15 cytokine-receptor complex (PDB ID: 4GS7), which signals in a trans manner. **d** The structure of the signaling complex of dimeric cytokines, exemplified by IFNγ (PDB ID: 6E3K)

The γc family of cytokines, including IL-2, IL-4, IL-7, IL-9, IL-15, and IL-21, holds a crucial position in lymphocyte development, growth, and survival, impacting immune responses across a spectrum of conditions, from cancer to immunodeficiency, allergies, and autoimmune disorders.^217,239,240^ The γc gene is located on the X-chromosome and expressed in the majority of lymphocyte populations. Over 200 different mutations in γc have been identified in individuals with X-linked SCID.^241–244^ The structure of IL-2 in complex with IL-2Rα, IL-2Rβ, and γc is the first signaling complex structure solved for γc cytokines.^245,246^ The crystal structure reveals a quaternary complex consisting of one copy each of IL-2, IL-2Rα, IL-2Rβ, and γc (Fig. 5a). Both IL-2Rβ and γc feature two disulfide bonds in the N-terminal domain (D1) and a ‘W-S-X-W-S’ motif in the C-terminal domain (D2). In IL-2Rβ, the D1 and D2 domains are linked by a helical linker and exhibit a bend at approximately 90°, while in γc, the D1 and D2 domains are bent at around 120°. IL-2Rβ and γc interact with distinct sites on IL-2; specifically, IL-2Rβ engages with helix 1 and helix 3, while γc interacts with helix 1 and helix 4. IL-2Rα binds to IL-2 at a third site, formed by helix 2 and two loops, positioned atop the quaternary complex.^245,246^ By contrast to the cytoplasmic domains of IL-2Rβ and γc, which associate with signaling kinases JAK1 and JAK3, the cytoplasmic domain of IL-2Rα is minimal, containing only 13 amino acids and does not actively participate in signaling. Despite IL-2Rα lacking direct contact with IL-2Rβ or γc, its primary function is to enhance the affinity of IL-2 to IL-2Rβ, and γc.^215,244,247^ While IL-2 alone binds to IL-2Rβ with a K_D_ of approximately 100 nM, the IL-2:IL-2Rα complex exhibits significantly higher affinity, with a K_D_ of around 30 pM when binding to IL-2Rβ.^248,249^ It has been proposed that upon IL-2Rα binding to IL-2, a conformational change occurs in IL-2, rendering it more favorable for subsequent binding with IL-2Rβ and γc, thereby augmenting the binding affinity.^245^ The signaling IL-2-receptor complex exists in two forms: a high-affinity form (10 pM) comprising IL-2Rα, IL-2Rβ, and γc, and an intermediate-affinity form (1 nM) consisting of only IL-2Rβ and γc.^215,245,250^ The structure of the second form is not currently available in the PDB. However, the quaternary structure of an engineered IL-2 variant, exhibiting enhanced affinity to IL-2Rβ, has been resolved in complex with IL-2Rβ and γc (Fig. 5b).^251,252^ This structure closely resembles the quaternary structure of the wild-type IL-2-receptor complex (RMSD = 0.6 Å), except for the absence of IL-2Rα. Therefore, it is proposed that the intermediate-affinity form of the IL-2 signaling complex is similar to the high-affinity form but lacks the α receptor.

Despite having limited sequence similarity (19% identity), IL-15 shares the same β and γ receptors as IL-2, with the only difference lying in the α receptor. In contrast to IL-2Rα, which possesses two Sushi domains, IL-15Rα only has one. The function of IL-15Rα is akin to that of IL-2Rα, enhancing the binding of the cytokine to the receptor. When the free IL-15 binds to IL-2Rβ, it has a K_D_ of 438 nM, the IL-15:IL-15Rα complex binds to IL-2Rβ with a K_D_ of 3 nM, resulting in approximately a 150-fold increase in affinity over free IL-15.^253,254^ While IL-2 signaling occurs in a cis manner, IL-15 signaling operates in a trans manner. The crystal structure of IL-15 signaling complex was solved by Aaron Ring and colleagues, revealing that IL-2Rβ and γc engage with site 1 (helix 1 and 3) and site 2 (helix 1, helix 4, and loop 1) of IL-15, respectively, while IL-15Rα binds to the third site composed of helix 2, loop 1, and loop 2 (Fig. 5c).^253^ Comparing the structures of IL-2 and IL-15 in complex with their receptors reveals almost identical features (RMSD = 1.2 Å), except for the α receptors.

While the majority of cytokines in the JAK-STAT pathway are monomeric, certain cytokines, such as IFNγ, IL-10, and IL-26, exist as dimers, necessitating the involvement of two pairs of receptors to form a quaternary complex. They follow a unique binding model to form a cytokine:receptor 1:receptor 2 complex with a 2:2:2 stoichiometry.^255–257^ Taking IFNγ as an example, its natural receptors consist of IFNγR1 and IFNγR2, both featuring a single CHR module in the extracellular domain. IFNγ binds to IFNγR1 with high affinity but to IFNγR2 with low affinity. While the structure of the IFNγ:IFNγR1 complex was reported in 1995, structures for either IFNγ:IFNγR1:IFNγR2 or IFNγ:IFNγR2 have not been available until 2019 when Christopher Garcia’s group reported the crystal structure of the complete signaling complex (Fig. 5d).^257^ In their study, protein engineering strategies were employed to enhance the affinity between the cytokine and receptor, resulting in a stable cytokine-receptor complex that yielded quality crystals for the structure determination. The complete 2:2:2 IFNγ receptor complex exhibits a star-shaped structure with a two-fold symmetry imposed by the IFNγ homodimer. The structure reveals six total interaction sites: two site 1 interfaces shared between IFNγ and IFNγR1, two site 2 interfaces shared between IFNγ and IFNγR2, and two site 3 interfaces shared between IFNγR1 and IFNγR2. The dimeric IFNγ is held by the cytokine binding regions from both receptors. While the C-terminal domains of the same type of receptor are approximately 70 Å apart, the C-terminal domains of IFNγR1 and IFNγR2 pairs are in close proximity, having protein contact with each other, which is critical for signaling.^257^

gp130, a pivotal member of the ‘tall’ class of cytokine receptors, serves as a shared receptor for various cytokines, including IL-6, IL-11, IL-27, IL-31, ciliary neurotrophic factor (CNTF), cardiotrophin-like cytokine factor 1 (CLCF1), leukemia inhibitory factor (LIF), oncostatin M (OSM), and cardiotrophin-1 (CT-1) (Table 1). These cytokines regulate a variety of complex biological processes, including hematopoiesis, immune response, inflammation, proliferation, differentiation, mammalian reproduction, cardiovascular action, and neuronal survival.^258–260^ gp130 is characterized by its substantial size, featuring an extracellular portion comprising six β-sandwich domains: a single Ig domain at the “top” (D1), followed by one CHR module (D2 and D3), and three FnIII domains (D4–D6), extending towards the cell membrane. Both the CHR and Ig domains play a crucial role in full receptor activation.^25,220,261–263^ The binding model of gp130 cytokines follows three distinct patterns.

**Fig. 6:**
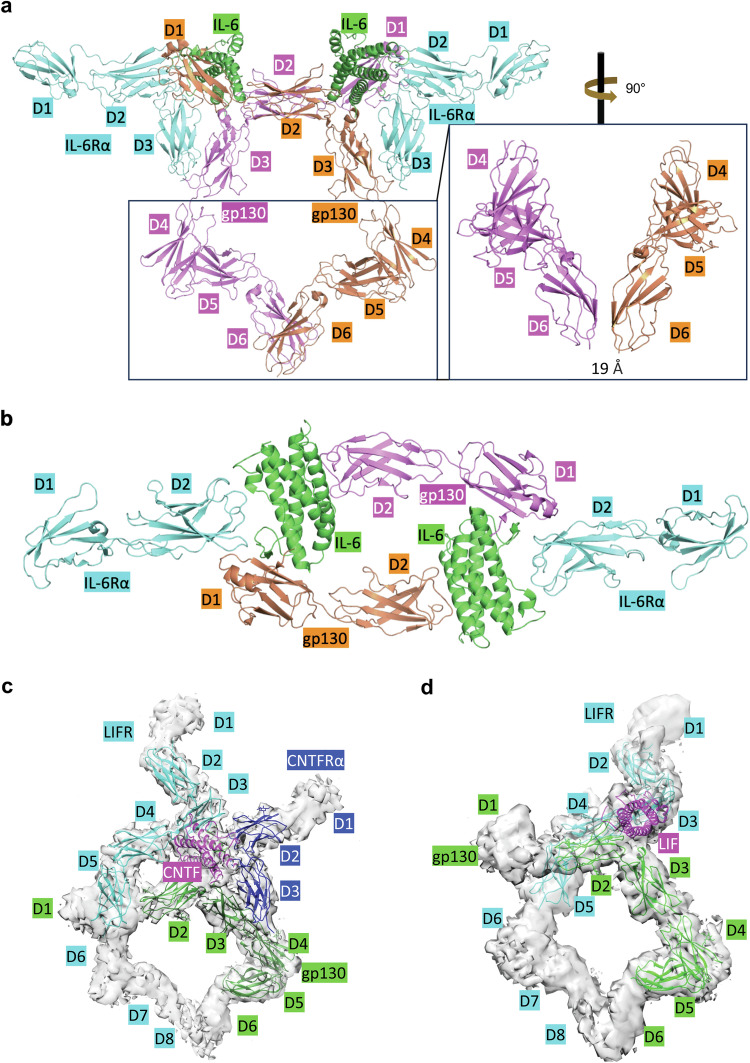
Structures of gp130 family cytokine-receptor complexes are illustrated, with domains labeled as letter ‘D’ plus a number, and the text highlighting correlating with the color of each domain. **a** The cryo-EM structure of the IL-6 signaling complex (side view, PDB ID: 8D82), comprising IL-6 (green), IL-6Rα (cyan), and two molecules of gp130 (magenta and orange). A bend between D4 and D5 of gp130 brings D6 close to each other at a distance of approximately 19 Å. **b** The top view of the IL-6 signaling complex (the same PDB ID as a). To maintain clarity, D3 of IL-6Rα and D3-6 of gp130 are not shown. **c** The cryo-EM structure of CNTF (magenta) in complex with CNTFα (blue), LIFR (cyan), and gp130 (green) (PDB ID: 8D74, EMDB ID: EMD-27229). Due to the cryo-EM density map quality, only part of the molecules has been modeled. **d** The cryo-EM structure of LIF (magenta) in complex with LIFR (cyan) and gp130 (green) (PDB ID: 8D6A, EMDB ID: EMD-27221), with only part of the molecules have been modeled for the same reasons as the CNTF signaling complex

The first pattern is exemplified by the IL-6 and IL-11 cytokine-receptor complexes, both consisting of two copies of cytokines, α receptors, and gp130s with a 2:2:2 stoichiometry (Table 1).^220,264^ The intracellular domains of IL-6Rα and IL-11Rα do not directly bind with JAKs or recruit STATs. Instead, their main role is to support gp130 in the formation of a functional cytokine-receptor complex. In certain instances, a soluble form of IL-6Rα, which includes the extracellular segment of the receptor, can bind to IL-6 with a comparable affinity to membrane-bound IL-6R. This interaction allows the soluble form of the receptor to facilitate the formation of a functional complex with gp130, leading to a process known as IL-6 trans-signaling.^265–268^ In this complex, the intracellular domains of gp130 interact with JAKs, executing the mission of transmitting information. Recently, Yi Zhou and colleagues solved the cryo-EM structure of the complete extracellular domain of gp130 in complex with IL-6 and IL-6Rα, offering valuable insights into IL-6 signaling (Fig. 6a).^220^ The structure reveals that the IL-6:IL-6Rα:gp130 exhibits two-fold symmetry, with cytokine-receptor interaction occurring at the top of the receptors. The CHR (D2 and D3) of IL-6Rα interacts with IL-6 at site 1, consisting of helix 1, helix 4 and loops, while D1 connects to D2 without contacting the cytokine. D3 of IL-6Rα interacts with D3 of gp130, contributing to the formation of IL-6:IL-6Rα:gp130 complex.^220^ By contrast, the CHR (D2 and D3) of gp130 engages with site 2 (helices 1 and 3) of one IL-6 molecule. Additionally, D1 of gp130 establishes contact with another IL-6 molecule, playing a crucial role in connecting two IL-6:IL-6 Rα:gp130 complexes. This interaction is pivotal for the formation of a symmetrical, interlocked structure within the hexamer of the IL-6 signaling complex (Fig. 6b).^220^ Although D4, D5, and D6 of gp130 are not involved in cytokine binding, they are structurally crucial for connecting to the transmembrane and intracellular domain of the receptor. While the distance between D3 of gp130 pair is ~80 Å, a sharp bend between D4 and D5 brings the “bottom” centers of the two gp130 juxtamembrane domains (D6) to ~19 Å apart (Fig. 6a).^220^ Even though there is no direct contact with the D6 of each gp130, the close distance might be sufficient to facilitate the triggering of signaling. In a separate study, Riley Metcalfe and colleagues solved the structure of IL-11 in complex with IL-11Rα and gp130 using X-ray crystallography and cryo-EM, demonstrating a cytokine-receptor binding pattern similar to that of IL-6.^264^

The second pattern of gp130 cytokine binding is exemplified by CNTF, CT-1, IL-27, and CLC (Table 1).^220,269,270^ The functional cytokine-receptor complex for these cytokines comprises three receptors: one α receptor, which participates in the formation of the functional complex but does not transmit information; one molecule of LIFR; and one molecule of gp130. Both the LIFR and gp130 bind with JAKs and are involved in information transduction. The cryo-EM structure of human CNTF signaling complexes was solved, with the most of the domains being modeled (Fig. 6c).^220^ While some domains were not modeled due to the low resolution of the cryo-EM density map, preventing atomic modeling of the complete structure, the overall shape observed in the electron density map indicates that CNTF signaling complexes may share a similar mechanism with the IL-6-receptor complex. A bend between D6 and D7 of LIFR is observed, reminiscent of the bend between D4 and D5 in gp130 (Fig. 6c).^220^ This arrangement brings the juxtamembrane domains of the cytokine-receptor complex close together, a feature potentially crucial for signaling.

The LIF, OSM, and IL-31 signaling complexes represent the third pattern of gp130 cytokine binding (Table 1). These cytokines lack α receptors, therefore the formation of the signaling complex has to rely on the β receptors and gp130.^220,271,272^ The structure of LIF signaling complex was determined using cryo-EM, with domains for cytokine-receptor interaction modeled (Fig. 6d).^220^ The overall structure of the LIF signaling complex closely resembles that of CNTF, with the notable absence of α receptors. Similarly, a distinct bend is observed in the cryo-EM density map, pinpointing the location of D6 and D7 of LIFR. Following this observed bending trend, the juxtamembrane domains of the receptors can maintain close proximity, a critical requirement for effective signaling.

Given the cytokine receptor recognition models, there is an ongoing controversy regarding how ligand binding to the extracellular portion of the receptor brings about the necessary interactions between the receptor’s cytoplasmic domains for signaling to occur. The controversy is whether the resting state of the receptor comprises individual receptor proteins that diffuse separately and come together into a complex only upon ligand binding or exist as preformed dimers that are activated by ligand-induced conformational changes.^25,273^ Recently, Stephan Wilmes and colleagues used dual-color single-molecule fluorescence microscopy in combination with posttranslational cell surface labeling to quantify the dimerization of three prototypic class I cytokine receptors in the plasma membrane of living cells, providing solid evidence in support of the ligand-induced receptor dimerization theory.^274^

### Molecular mechanism for JAK activation

**Fig. 7:**
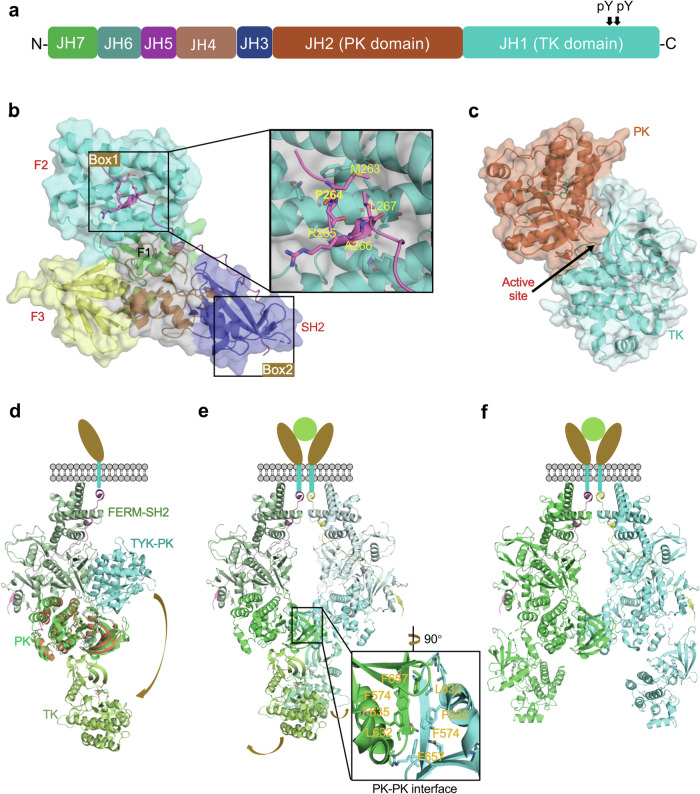
Receptor recognition and activation of JAKs. **a** Schematic diagram showing the domain organization of JAKs. **b** Interaction between JAKs and receptors, illustrated by the structure of the intracellular domain of IFNλR1(magenta) in complex with the FERM-SH2 domain of human JAK1 (PDB ID: 5L04). The two primary binding regions from the intracellular receptor domain correspond to the ‘box 1’ and ‘box 2’ motifs, which bind to the F2 subdomain and SH2-like domain, respectively. **c** Structure of the PK and TK domains from human TYK2 (PDB ID: 4OLI), representing an autoinhibitory state. **d** The structure of full-length mouse JAK1 (mJAK1), derived from the mJAK1 pair (shown in **e**). When the PK domains of the TYK2 PK-TK structure (shown in **c**) and mJAK1 are superimposed, the TYK2 TK domain is found closer to the membrane, representing an autoinhibited state. Dimerization induced by cytokine binding likely causes a conformational change in the TK domain, relieving it from the autoinhibited state. **e** Dimeric structure of the mJAK1 complex (without nanobody stabilization, PDB ID: 8EWY), where PK domains form a zipper-like structure held together by a hydrophobic cluster of phenylalanine residues and an antiparallel β-sheet. The V657F mutation (equivalent to the pathogenic V617F mutation in human JAK2 PK domain) enhances the PK-PK interaction. TK domains also form a protein-protein interface in this structure, with active sites facing each other. **f** Dimeric structure of the mJAK1 complex (with nanobody stabilization, PDB ID: 7T6F), where TK domains rotate and the active sites face outward, proposed as an active state of JAK

Unlike receptors for growth factors like insulin or epidermal growth factor (EGF) that have intracellular domains possessing tyrosine kinase activity on the same polypeptide chain, receptors for the JAK-STAT pathway cytokines have no intrinsic enzymatic activity. Instead, the intracellular domains of these receptors are constitutively associated with tyrosine kinases of the JAK family.^13,25,275,276^ There are four JAKs–JAK1, JAK2, JAK3, and TYK2; each associates with different cytokine receptors. JAKs have seven JAK homology (JH) domains (Fig. 7a).^13,39^ Starting from the carboxyl terminus, the first domain is JH1, also known as the tyrosine kinase (TK) domain. JH1 is approximately 275 amino acids in length and encodes a protein kinase that constitutes the catalytic domain responsible for phosphorylating substrate molecules. JH2, also referred to as the PK domain, shares structural similarities with the TK domain but is believed to be a catalytically inactive. Nonetheless, studies have shown that the PK domain of JAK2 exhibits some activity by phosphorylating two negative regulatory sites in JAK2 (S523 and Y570). The PK domain plays a crucial role in regulating the activity of the kinase domain.^277,278^ JH3 and JH4 in JAKs correspond to SH2 domains, while JH5, JH6 and JH7 constitute the “four-point-one, ezrin, radixin, moesin” (FERM) domain. The SH2 and FERM domains in JAKs work together to facilitate cytokine receptor recognition and binding by JAKs.^279,280^

The intracellular domains of cytokine receptors play a crucial role in binding and recruiting JAKs and STATs, complementing the extracellular part responsible for cytokine recognition.^279,280^ Within these intracellular domains, conserved regions called ‘box 1’ and ‘box 2’ are essential for the interaction with JAKs. The box 1 motif, located proximal to the cell membrane, is a proline-rich sequence, while the hydrophobic box 2 motif is separated from box 1 by a variable number of residues. Mutations within the box 1 or box 2 motifs have been found to disrupt the binding of JAKs to cytokine receptors and consequently affect downstream signaling events. The four JAK family members share a conserved domain organization consisting of FERM and SH2 domains at the N-terminus, forming a ‘FERM-SH2 holodomain’ that is crucial for binding to the cytoplasmic domain of cytokine receptors.^279–281^ Several crystal structures of the FERM-SH2 holodomain in complex with JAK-binding motifs of intracellular domains of cytokine receptors have been solved, including JAK1-FERM-SH2 holodomain bound to IFNλR1-box 1, JAK2-FERM-SH2 holodomain bound to EPOR-box 1-box 2, TYK-FERM-SH2 holodomain bound to IFNAR1-box 2, and IFNλR1-box 1-box 2.^279,282,283^ These structures have demonstrated that the FERM-SH2 module consists of four subdomains; ubiquitin-like F1 domain, acyl-CoA-binding protein-like F2 domain, and pleckstrin homology domain-like F3 domain form a tri-lobed FERM domain, and the SH2-like domain packs against the F1 and F3 domains, forming the base of the Y shape structure (Fig. 7b). The F2 domain, characterized by a large basic patch, suggests its association with the plasma membrane of cells. It primarily engages in binding the membrane-proximal box 1 motif of the receptor. On the other hand, the SH2-like domain interacts with the box 2 motif. Together, the box 1 and box 2 motifs form an elongated epitope spanning approximately 85 Å, with extensive interactions burying over 1000 Å^2^.^25,33,277,279,282–285^ The contribution of box 1 and box 2 motifs to the overall affinity may vary among different receptors. For instance, in the IFNλ receptor, box 1 provides the majority of the affinity, while the addition of box 2 further enhances it by 10-fold. By contrast, the binding of TYK2 to IFNAR1 appears to be predominantly driven by box 2.^279,280^ Importantly, the key features of JAKs binding to box 1 and box 2 motifs are conserved, suggesting a similar JAK-receptor recognition mechanism across different JAKs.^279,282,284^

In JAKs, the PK and TK domains are arranged in a tandem fashion that allows for functional coordination between the two domains in JAK signaling.^277,286^ Despite the PK domains of JAKs lacking kinase activity, they play a crucial role in regulating these proteins via an autoinhibitory mechanism. One important finding is that the deletion of the PK domain results in an increase in the basal level of kinase activity, but prevents further enhancement of activity in response to cytokines. This underscores the significance of the PK domain in maintaining the precise balance of JAK activity and fine-tuning of the signaling process.^287,288^ The importance of the PK domain was further highlighted when a specific V617F point mutation was discovered in human JAK2; the mutation is associated with the development of various MPNs.^52,53^ The crystal structure of the two-domain PK-TK module of TYK2 has provided insights into the mechanism of autoinhibition by the PK domain.^286^ The interaction between the PK and TK domains of TYK2 is primarily facilitated by their respective N-terminal lobes, forming a distinctive angle. This interaction buries a significant surface area of approximately 1,500 Å^2^, characterized by hydrophobic and polar contacts. It has been proposed that this autoinhibitory mechanism involves head-to-head dimerization of the PK-TK domains, potentially obstructing substrate binding and catalytic activity (Fig. 7c).^286^ Experimental evidence comparing the kinase activity of the PK-TK domain construct with the kinase domain alone supports this mechanism, as the TK domain alone exhibits higher activity. The PK-TK interaction that maintains JAKs in an inactive state may serve as a regulatory mechanism to prevent excessive signaling in the absence of cytokine binding to receptors.^289–291^

The recent determination of the dimeric structure of full-length mouse JAK1 (mJAK1) complexed with the intracellular box 1-box 2 segment of IFNλR1, led by Christopher Garcia’s group, sheds light on the mechanism of JAK activation (Fig. 7d).^24,285^ To obtain a stable and non-aggregated complex suitable for cryo-EM, the researchers employed several techniques. They replaced the transmembrane domains of the receptor with the homodimeric GCN4 leucine zipper fused to the IFNλR1 box 1-box 2, creating a soluble mimic of a dimerized receptor. Biochemical studies demonstrated that the complex formed by the IFNλR1 intracellular domains containing the leucine-zipper peptide and full-length wild-type mJAK1 or mJAK1-V657F (equivalent to V617F in human JAK2) can mimic the natural receptor dimer complex. Additionally, the complex was further stabilized by expressing mJAK1 with a C-terminal nanobody epitope tag (BC2T) and using a dimeric BC2 nanobody to reduce conformational heterogeneity. Ultimately, using 3D reconstruction, the structures of the JAK1-IFNλR1 complex with and without nanobody stabilization were solved at resolutions of 3.6 Å and 5.5 Å, respectively.^24,285^

Both structures exhibit C2 symmetry and contain two copies of the box 1-box 2 peptide and mJAK1 (Fig. 7e and f).^24,285^ The complex reveals that the box 1-box 2 peptide binds to the FERM-SH2 domain of JAK1 along an extended groove through its box 1-box 2 motifs, which is similar to the crystal structure of the JAK1-FERM-SH2:box 1-box 2 complex.^24,283,285^ The FERM-SH2 domains sit above the inward-facing PK domains, which form a head-to-head dimer at the center of the complex. The PK domains are in an inactive conformation, characterized by a closed activation loop and an outward rotation of the catalytic glutamate. The mJAK1 homodimer is formed by the interaction of the SH2-PK linker and PK N-terminal lobes from individual mJAK1 monomers, which are held together by a hydrophobic cluster of phenylalanine residues and an antiparallel β-strand (Fig. 7e).^24,285^ The V657F mutation enhances the structural and hydrophobic complementarity of the wild-type dimer interface, promoting dimerization and potentially facilitating ligand-independent activity of this clinically relevant mutation in the PK domain (V617F in human JAK2).^24,52,285^ When the PK domain from a previously reported crystal structure of the autoinhibited TYK2 PK-TK domain is superimposed onto the PK domain from one chain of the full-length mJAK1 dimer complex, it reveals a conformational change in the TK domain from a closed, autoinhibited state to an open conformation (Fig. 7d). The closed conformation is incompatible with JAK dimerization, as it results in a steric clash within the JAK dimer.^24,285,286^ However, previous negative-stain electron microscopy imaging of a JAK1 monomer has indicated that it can adopt a range of conformational states, including a compact closed state and an extended open state.^24,285,292^ AlphaFold 2 predictions of the monomeric structures of the four JAK proteins have also revealed two distinct configurations. One configuration shows a PK-TK interaction similar to that observed in the crystal structure of TYK2 PK-TK, representing the autoinhibited state of a JAK. The other configuration closely resembles the PK-TK interaction observed in the cryo-EM structure of homodimeric mJAK1, representing an open state. When utilizing AlphaFold 2 to predict PK-TK dimeric structures for different physiological wild-type JAK dimers, it was observed that the highest-scoring models consistently showed similarities to the PK-TK (cis), PK-PK (trans), and TK-TK (trans) interfaces observed in the 5.5 Å resolution cryo-EM structure of mJAK1. Therefore, it can be proposed that there is a spontaneous transition between these two states for the monomeric JAK, whereby the autoinhibited state is disrupted when two copies of JAK come close together, leading to the formation of the open state.^24,285,286,292^

Comparing the structures of the nanobody-stabilized mJAK1 and the nanobody-free mJAK1, it is evident that the two structures are highly similar, except for the conformation of the TK domain (Fig. 7e and f).^24,285^ In the nanobody-free structure, a protein-protein interface is formed in the TK C-terminal lobes, resulting in a little buried surface area of 783 Å^2^ (Fig. 7e). The active sites of the TK domains are positioned face to face in this configuration. By contrast, in the nanobody-stabilized structure, the TK domain undergoes an approximately 180° rotation, causing the active site to face outward, consequently disrupting the TK-TK interface (Fig. 7f). It was suggested that the nanobody-free model represents a trans-activated state of JAK, while the nanobody-stabilized structure represents the active state. By employing the 3D variability analysis method, the intermediate states between these two structures can be traced, indicating the flexibility of the TK domain and the potential existence of multiple conformational states during JAK activation. During the transition between these states, the auto-phosphorylation of two tyrosine residues within the activation loop of the JAK kinase domain occurs, followed by the phosphorylation of the intracellular domain of the receptors, thereby creating docking sites for downstream STAT molecules to initiate signaling.^24,285^

### STAT phosphorylation and transcription initiation

**Fig. 8:**
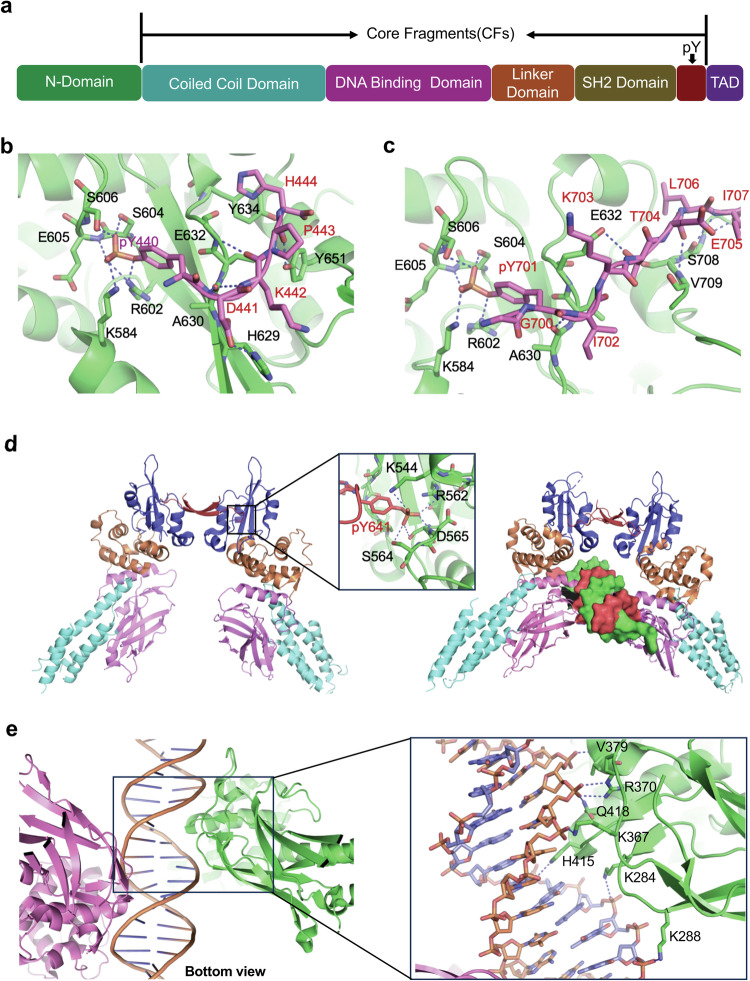
Phosphorylated tyrosine recognition and DNA binding by STATs. **a** Schematic diagram illustrating the domain arrangement in STATs. **b** Interaction between STATs and receptors, occurring during the STATs recruitment step; illustrated by the structure of a phosphopeptide (^440^pYDKPH^444^, shown in magenta color and labelled in red color) derived from the α-chain of the human IFNγ receptor in complex with the SH2 domain (shown in green color and labelled in black color) of STAT1 (PDB ID: 1YVL). The phosphorylated tyrosine forms extensive hydrogen bond networks with the SH2 domain, contributing significantly to the binding affinity of the phosphopeptide. **c** Detailed structure of the SH2 region of phosphorylated STAT1 (PDB ID: 1BF5). The phosphorylated tyrosine and its surrounding residues from its partner (shown in magenta color and labelled in red color) bind to the SH2 domain (shown in green color and labelled in black color), similar to the interaction of receptor and unphosphorylated STATs (as shown in b). **d** The overall structure of the phosphorylated STAT dimer without DNA (left, PDB ID: 4Y5U) and with DNA (right, PDB ID: 4Y5W), exemplified by STAT6. **e** Molecular details of STAT-DNA interaction, illustrated by using the structure of the STAT6-DNA complex (PDB ID: 4Y5W)

The STAT family has seven members (STAT1, STAT2, STAT3, STAT4, STAT5A, STAT5B, and STAT6), which share a common structural organization of around 600–850 amino acids. From the N- to the C-terminus, these proteins contain an N-terminal domain, a coiled-coil domain, a DNA binding domain, a linker region, an SH2 domain, and a transactivation domain (TAD) (Fig. 8a).^26,293^ The recruitment of different STAT family members can be mediated by a single phosphorylated tyrosine (pY), with varying affinities dictated by the surrounding sequence on the intracellular domain of the receptor; the motifs pYXXP, pYXXQ, pYXXL, and pYXXF are associated with STAT1, STAT3, STAT5, and STAT6 recruitment, respectively. Some receptors have multiple STAT binding sites; for instance, gp130 contains four STAT3 binding motifs, while other receptors, such as IFNγR, feature only a single STAT binding site.^13,293–297^ The interaction between the receptor motif and STATs is mediated by the SH2 domain of STAT, as demonstrated by the crystal structure of unphosphorylated STAT1 in complex with a phosphopeptide (^440^pYDKPH^444^) derived from the α chain of the human IFNγ receptor.^298^ In this structure, it is observed that pY440 of the peptide interacts with K584, R602, S604, E605 and S606, as well as the phosphate binding loop of the SH2 domain. Additionally, peptide residues D441 (pY + 1) and H444 (pY + 4) form hydrogen bonds with H629 and Y634 of the SH2 domain, respectively (Fig. 8b).

It is believed that STATs pre-exist, prior to phosphorylation, in the form of homodimers, heterodimers, or higher oligomeric states, although they are not in a conformation ready for DNA binding.^298–301^ After being recruited by the intracellular domain of the receptor, STATs undergo phosphorylation, and the phosphorylated tyrosine, along with its surrounding residues, competes with the receptor for binding to STATs. This competition results in the release of STATs from the receptor, leading to the formation of a STATs dimer that is translocated into the cell nucleus and prepared for DNA binding.^298,302,303^ In certain scenarios, STATs can also exert their effects by forming complexes with other transcription factors or regulatory proteins, thereby finely regulating gene transcription. For instance, downstream of type I and type III IFNs, STAT1 and STAT2 can associate with interferon regulatory factor 9 (IRF9) to form the IFN-stimulated gene factor 3 (ISGF3), which plays a crucial role in imparting functional specificity to cytokine signaling.^7,304,305^ The structures of phosphorylated STATs have been solved both in the presence and absence of DNA binding (Fig. 8d).^26,293,306^ The monomers in the dimeric STATs are associated through a 2-fold symmetry axis. The interaction between the monomers is primarily mediated by the SH2 domains, which undergo an intimate exchange of C-terminal segments. These C-terminal segments extend from the SH2 domains of each monomer, bind to the SH2 domain of the other monomer, and form an antiparallel β-strands arrangement with each other. They then return to interact with their respective parent SH2 domain. This interaction between the SH2 domains creates a closed embrace, firmly securing the STAT dimer onto DNA. The core of the SH2 domain is composed of an antiparallel β-sheet flanked by two α helices, while the phosphorylated tail segment, originating from the other monomer in the dimer, binds in an extended conformation perpendicular to the β-strands.^26,293,306^ The recognition of the phospho-tyrosine residue by the SH2 domain involves the interaction of the phosphate group with conserved amino acids, similar to their counterparts observed in the crystal structure of unphosphorylated STAT1 complexed with an IFNγ receptor phosphopeptide (Fig. 8b and c). This similarity highlights a conserved mechanism for phospho-tyrosine recognition in STATs.^26,298^ Interestingly, even in the absence of DNA, the structures of phosphorylated STATs exhibit a letter V-like shape, resembling the conformation observed in the STAT-DNA complex, and suggesting that phosphorylation induces a conformational change that prepares the protein for DNA binding (Fig. 8d). The pre-formed conformation of phosphorylated STATs suggests that phosphorylation plays a critical role in priming the protein for DNA recognition.^38,293,306^ The DNA binding domain of STATs, characterized by an immunoglobulin fold, plays a critical role in DNA recognition. The β-strands within this domain run parallel to the major axis of the domain, which is oriented perpendicular to the direction of the DNA axis. As a result, the loops located at one end of the β-sandwich face the DNA. In the STAT6:DNA complex, a helix, along with the turn at the C-terminal of the DNA binding domain of STAT6, inserts into the major groove of the DNA (Fig. 8e). Specifically, H415 on this helix forms a hydrogen bond with the guanine base, playing a crucial role in the STAT-DNA interaction. Additionally, several charged amino acid residues such as lysine and arginine interact with the DNA by forming charge contacts with the phosphate groups of the DNA. Interestingly, a similar helix-turn structure that inserts into the major groove of the DNA is also observed in the STAT1-DNA complex structure. However, in this case, an asparagine-thymine interaction is observed instead of the histidine-guanine interaction seen in the STAT6 complex. Therefore, the helix-turn structure not only contributes to DNA binding but also determines the DNA sequence specificity.^26,293^

### A complete picture of the JAK-STAT signaling process

**Fig. 9:**
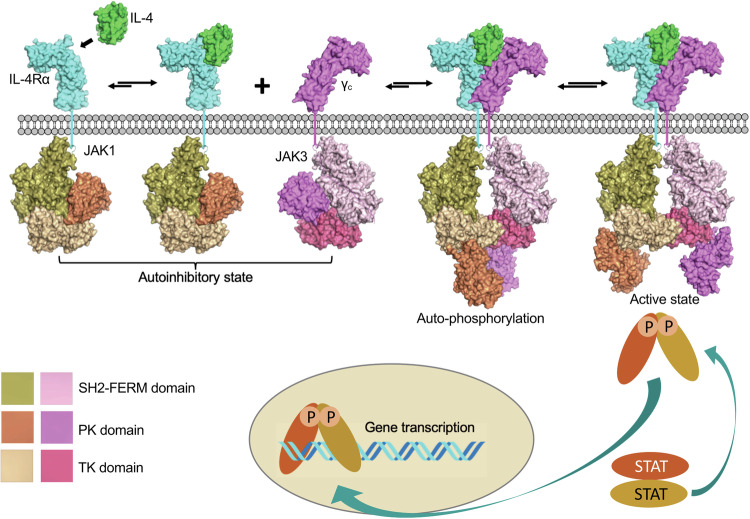
The signaling process of the JAK-STAT pathway is depicted using the IL-4:IL-4Rα:γc signaling complex as an example. The JAK1 and JAK3 models are homologous structures built based on the mJAK1 structure and the human TYK2 PK-TK structure

With the knowledge mentioned above, we can now paint a clearer picture of the signal transduction process for the JAK-STAT pathway. Here we use the first IL-4 signaling complex (IL-4:IL-4Rα:γc) as an example to demonstrate this process (Fig. 9). While this model may not fully capture all cytokine-receptor interaction patterns, it serves as a useful demonstration of how cytokine dimerization activates JAK pairs. Cytokine receptors pre-exist on the membrane of certain types of cells, and JAKs are associated with the C-terminal region of the receptor via their FERM-SH2 domains. Initially, the receptor-JAK complex exists separately, and the autoinhibition by the PK domain keeps the JAK enzyme inactive. Upon cytokine binding, the receptors undergo dimerization or oligomerization, leading to the proximity of two JAKs. This close proximity facilitates dimerization through protein-protein interactions involving the PK domains, resulting in the release of autoinhibition, auto-phosphorylation and activation of JAKs, phosphorylation of the receptor and the creation of docking sites for STATs that enables their recruitment. Once the STATs are phosphorylated, they are translocated to the cell nucleus and undergo conformational changes, enabling them to bind to specific DNA sequences and trigger gene transcription.

Advancements in the structural biology of the JAK-STAT pathway not only expand our understanding of how this pathway functions at the molecular level but, more significantly, provide fundamental knowledge for therapeutic development and offer new opportunities for the pharmaceutical industry.^19,35,169^ The rise of cytokine engineering (a topic we will discuss in subsequent sections), which is primarily based on our understanding of the structural biology of the JAK-STAT pathway, in recent years promises to revolutionize the pharmaceutical industry.

### Cytokine engineering

While there are several cytokines approved by the FDA as therapeutics, they represent only a small fraction of the total number of cytokines. It is worth noting that no new cytokines have been approved as therapeutics in the past two decades.^193,198^ The development of cytokine therapeutics poses challenges due to their complex nature and interactions within the immune system. First, cytokines are small proteins, which makes them prone to a high renal clearance rate. As a result, they have a short half-life in the body, requiring frequent administration to maintain effective therapeutic concentrations. Second, cytokines are typically produced locally and act in specific cell types, tissues, or organs. However, cytokine drugs are generally administered systemically. This can result in inadequate concentrations if low doses are given or broad systemic effects if high doses are used. Last, cytokines exhibit pleiotropic effects, meaning they can act on multiple cell types and affect various biological functions. This poses a challenge, as it can lead to dose-limiting toxicities or limited efficacy when targeting specific pathways or cell types.^169,193,307,308^ Taking FDA-approved IL-2 as an example, although it has shown the ability to induce tumor regression, its effectiveness in improving patients’ overall survival is limited. This is primarily attributed to its dual functional properties, as it can act on both regulatory T (T_reg_) cells and effector T (T_eff_) cells, leading to conflicting effects. Furthermore, IL-2 can cause severe adverse effects when administered at high doses. In contrast, the most successful cytokine drugs to date, such as EPO, G-CSF, and GM-CSF, target receptors that are expressed in a more restricted subset of cells, such as hematopoietic cells.^169,193,197^ However, recent progress in structural biology within the JAK-STAT pathway, combined with advancements in protein engineering techniques, has opened new avenues in cytokine engineering. This advancement offers the potential for tailored cytokine therapeutics with improved safety and efficacy. Many cytokines previously deemed unsuitable for drug development are now demonstrating promise in clinical trials.^169,193,307,308^

One common cytokine engineering technique involves conjugating cytokines with polyethylene glycol (PEG), which can improve their pharmacokinetics and pharmacodynamics. PEG conjugation (PEGylation) offers several advantages for cytokines. Firstly, PEGylation helps reduce renal clearance, extending the half-life of cytokines in the bloodstream. Additionally, it can protect the cytokine from proteolytic enzymes, ensuring its stability.^309,310^ In fact, several cytokine drugs that have received FDA approval are PEG-modified recombinant cytokines, including Pegasys (PEG-IFNα-2a), Sylatron (PEG-IFNα-2b), Mircera (PEG-EPO), and Neulasta (PEG-G-CSF); they have demonstrated improved pharmacokinetic profiles.^309^ For instance, the PEGylation of IFNα has shown remarkable results. It substantially increases the serum half-life of IFNα from 3–8 hours to 65 hours and significantly reduces the clearance rate by over 100-fold. This prolonged activity allows for a once-a-week administration, compared to the thrice-weekly dosing required with unconjugated IFN, in the treatment of chronic hepatitis C.^311,312^ However, there are limitations to PEGylation of cytokines that should be considered. For instance, the modification of side chains on surface amino acids of the cytokine may introduce perturbations to the protein structure, resulting in changes in binding affinity to the receptor and subsequent effects on biological activity. In some cases, PEGs can shield the binding site on the cytokine, leading to a blockage of its biological activity. Additionally, untargeted conjugation of PEG on the amino acids on the protein surface can result in heterogeneous products. However, these drawbacks can be addressed by utilizing site-specific PEG conjugation methods.^309,310^ For example, certain amino acids, such as cysteine, may be less commonly present on the surface of cytokines. Cysteines can form stable thioethers or disulfides with PEG-cysteine reagents, enabling specific protein conjugation. Introducing cysteines at specific positions on the cytokine surface can be an effective approach for targeted PEG conjugation.^307–309,313^ Additionally, other methods, such as fusing cytokines to albumin or the Fc domain of an IgG, have been explored to reduce renal clearance and extend the half-life of cytokines.^307,308^

To mitigate the side effects associated with systemic administration of cytokines, various approaches can be implemented to enhance the targeted delivery of cytokines to the disease site. One such approach involves the use of antibody-cytokine fusion proteins, known as immunocytokines. These fusion proteins combine the disease-targeting capabilities of specific antibodies with the immunomodulatory properties of cytokine payloads. The antigen-binding domain of an immunocytokine is designed to direct cytokines to a specific tissue or cell type.^314,315^ Another approach is conditional activation. For instance, proteases are often upregulated in many tumors and play significant roles in cancer development and progression, by facilitating extracellular matrix remodeling, tumor invasion, and metastasis. In this approach, the cytokines are initially masked and rendered inactive. Upon reaching the TME, proteases cleave the linker between the cytokines and the masking molecule, leading to the release of active cytokines specifically at the tumor site.^307,308,316,317^ Other strategies, such as local injection or advanced drug delivery systems, can also be employed to increase cytokine concentration specifically at the site of the disease.^307,308^

The pleiotropic nature of cytokines poses a significant challenge in the development of cytokine therapeutics, as one cytokine has the ability to activate multiple cell types within a tissue or multiple signaling pathways within a cell. However, for therapeutic purposes, it is often necessary to selectively activate specific cell types or signaling pathways. To mitigate the side effects in cytokine therapy, one key approach is to decouple the pleiotropy of the cytokine, which involves narrowing down the downstream signals triggered by a specific cytokine. While the downstream signals in the JAK-STAT pathway are influenced by various factors, including the type of cytokine, its concentration, receptor composition, the affinity of cytokine-receptor interactions, the distribution of cytokine receptors on cells, and the presence of other cytokines, the primary step in implementing cytokine pleiotropy decoupling is to modify the affinity between a particular cytokine and its receptor.^169,318^ In the JAK-STAT pathway, many cytokines engage in a two-step process to form heterodimer receptor complexes. Initially, they bind to their specific cytokine receptors, typically with high affinity, which can determine the EC_50_ (half-maximal effective concentration) of the cytokine. Subsequently, the cytokine-receptor complex associates with the shared receptor, whose affinity influences the E_max_ (maximal effect) of the signaling response.^25,169,245,274,319^ Certain cytokine-specific receptors are predominantly found on the surface of specific cell types. The affinity between the cytokine and its specific receptor is a crucial factor that influences the function of the cytokine, particularly when the cytokine is present at low concentrations. High affinity facilitates effective attraction of the cytokine to its receptor.^169,245,319^ On the other hand, the binding of a particular cytokine to the shared receptor involves competition with other cytokines that also bind to the same receptor. Consequently, the affinity between the cytokine and the receptor determines its competitiveness among other cytokines, thereby influencing the amplitudes of the signaling response.^13,169,319^ In addition, certain cytokines, such as type I IFNs, can bind to the same pair of receptors but trigger different downstream signals and exert distinct biological functions. While the local concentration of specific cytokines contributes to this discrepancy, the affinity between the cytokine and receptors also plays a crucial role in determining their functional outcomes.^320,321^ Furthermore, some cytokines, including IL-2, IL-4, IL-20, and IL-24, can engage with multiple receptor complexes. In these cases, the affinity of the cytokine for specific receptor complexes becomes crucial for achieving selective signaling and functional outcomes.^13,25,245^ By modifying the cytokine’s affinity to a particular receptor, it becomes possible to restrict its activity to a narrower range of cells or limit its effects to specific downstream signals. This approach offers the advantage of reducing the potential toxicity associated with cytokine pleiotropy and improving the overall safety profile of cytokine therapeutics.^169,319^ Advancements in protein engineering technology provide a toolbox for modifying cytokines to exhibit desired functions or signaling behaviors. One approach is directed evolution, where mutations are introduced into a gene of interest, followed by the use of selection methods, such as yeast and phage display, to obtain a protein with the desired function.^319,322^ Additionally, experimental determination of cytokine structures and cytokine-receptor complex structures, along with the use of accurate 3D models through artificial intelligence (AI)-based protein structure prediction approaches, enable us to make rational mutations on cytokines to alter their properties. These protein engineering techniques offer the potential to decouple the pleiotropic effects of cytokines and design them to have more specific and targeted actions.^25,169,322^

In the following section, we will explore specific examples of key cytokines to highlight the application of cytokine engineering for therapeutic development.

### IL-2 and IL-15

**Fig. 10:**
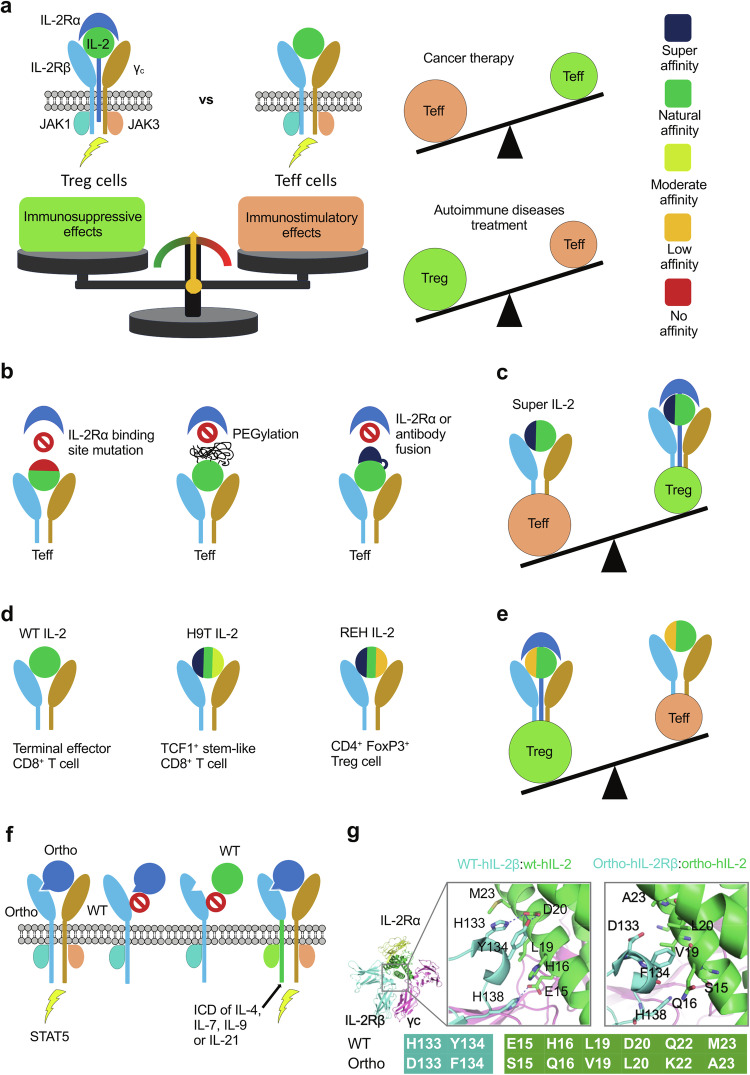
IL-2-based therapeutics development. **a** The immunostimulatory and immunosuppressive effects are balanced by IL-2. Decoupling the pleiotropic effects of IL-2 is essential for therapeutic purposes, utilizing its immunostimulatory effects for cancer therapy and its immunosuppressive effects for autoimmune disease treatment. **b** Strategies for decoupling the pleiotropy of IL-2 by disrupting IL-2Rα binding. **c** Strategies for enhancing the T_eff_ to T_reg_ cells ratio via ‘superkines’ (super IL-2) to modulate the immune response. **d** Wild-type IL-2 and IL-2 partial agonists, varying in their affinity to γc, can induce different cell fates. **e** Strategies for enhancing the T_reg_-to-T_eff_ cell ratio using IL-2 partial agonists, which have greater dependency on IL-2Rα. **f** The principle of orthogonal (ortho) IL-2 cytokine-receptor pairs. Ortho-IL-2 is an IL-2 mutant designed to exclusively bind to the ortho-IL-2Rβ:γc pair, while showing no affinity for the wild-type IL-2Rβ:γc pair. Similarly, Ortho-IL-2Rβ refers to an IL-2Rβ mutant specifically engineered to interact solely with ortho-IL-2, with no binding to the wild-type IL-2. Ortho-IL-2Rβ can be employed to generate chimeric orthogonal receptors capable of transducing signals for cytokines like IL-2, IL-9, or IL-21, by simply replacing the intracellular domain of ortho-IL-2Rβ. **g** Structural basis for designing human ortho-IL-2 and ortho-IL-2 receptor pairs

IL-2 is one of the key cytokines with pleiotropic effects on the immune system, balancing both immunostimulatory and immunosuppressive effects (Fig. 10a).^194^ As previously stated, there are two distinct receptor binding forms, with the first form (high-affinity complex) involving IL-2Rα, IL-2Rβ, and γc, and the second (intermediate-affinity form) solely involving IL-2Rβ and γc (Fig. 10a).^25,245,253^ IL-2Rα is a “private” receptor for IL-2 and is primarily expressed on T_reg_ cells, and to some extent on Langerhans cells, endothelial cells, and fibroblast cells. It does not directly transmit intracellular signaling; instead, its primary function is to facilitate the recruitment of IL-2 and enhance the binding affinity of the cytokine to the receptor complex, sensitizing signals to low cytokine concentration.^245,323^ The assembly of the high-affinity complex occurs sequentially, with IL-2 initially binding to IL-2Rα, which then facilitates the engagement of IL-2Rβ, and ultimately recruits the γc subunit to form the functional complex.^245^ It primarily initiates signaling cascades that lead to immunosuppressive effects, whereas the second binding form of the complex activates signaling cascades that are responsible for immunostimulatory effects.^194,245,323^ The divergent outcomes resulting from the differential affinity and composition of the IL-2 receptor complexes are often preferred for different therapeutic purposes. For instance, in the treatment of autoimmune diseases, immunosuppressive effects of IL-2 are desirable, while in cancer treatment, immunostimulatory effects are sought after (Fig. 10a).^194–197,324^

High dosages of IL-2 have been approved for cancer treatment, primarily because when the concentration is low, the majority of IL-2 likely binds to the IL-2Rα:IL-2Rβ:γc complex. Only when this complex becomes saturated, excess IL-2 can exert its immunostimulatory effects for tumor elimination.^195–197^ However, high doses of IL-2 can lead to significant side effects, including severe systemic toxicities like vascular leak syndrome, pulmonary edema, hypotension, acute renal insufficiency, and rarely myocarditis. Moreover, prolonged treatment with high doses of IL-2 may result in T cell exhaustion and cancer progression due to T_reg_ cells expansion.^195,325^ To address these issues, cytokine engineering approaches can be employed to modify IL-2 in a way that enhances the T_eff_-to-T_reg_ cell ratio.^326^ This approach is essential for reducing side effects, boosting the effectiveness of immunotherapy, and decreasing the immunosuppressive effects caused by T_reg_ cells. One simple and direct idea is to abolish the binding of IL-2 to IL-2Rα by introducing specific mutations at the IL-2Rα binding site. By disrupting the interaction between IL-2 and IL-2Rα, the corresponding IL-2 variant can exclusively bind to IL-2Rβ:γc complex to boost T_eff_ cells (Fig. 10b). Tania Carmenate and colleagues developed a human IL-2 mutant with enhanced antitumor efficacy and reduced toxicity compared to the wild-type IL-2.^327,328^ The mutant contains four mutations in the interface with IL-2Rα. While the IL-2 mutant efficiently induces proliferation of CD8^+^CD44^hi^ cells and NK1.1 cells similar to wild-type IL-2, it exhibits lower capacity to stimulate the proliferation of CD4^+^Foxp3^+^ T_reg_ cells. In various transplantable tumor models, the IL-2 mutant demonstrates superior antimetastatic effects compared to wild-type IL-2. Notably, when used at high doses in mice, the IL-2 mutant causes less lung and liver toxicity than wild-type IL-2.^327,328^ Christian Klein and colleagues reported the development of an IL-2-based immunocytokine, carcinoembryonic antigen (CEA)-IL2v, which selectively binds to the IL-2Rβ:γc complex. They initially introduced several mutations that abolished the binding of IL-2 to IL-2Rα. Subsequently, the mutant was fused to a CEA-specific antibody, which enhances pharmacokinetic properties and increases the therapeutic index by targeting CEA. Preclinical data demonstrated the therapy’s efficacy, especially when used in combination with an anti-PD-L1 antibody.^329^

Another approach, IL-2 with the binding site for IL-2Rα being shielded, can also boost T_eff_ cells, but not T_reg_ cells (Fig. 10b). This can be achieved in several ways. For example, the PEGylation of IL-2 specifically at the IL-2Rα binding site can effectively block the binding of IL-2 to IL-2Rα. Deborah Charych and colleagues developed NKTR-214, a PEGylated IL-2 with six PEG chains conjugated to lysine residues (K31, K34, K42, K47, K48, and K75) situated at or near the IL-2Rα binding site, thereby blocking the interaction with IL-2Rα. In a murine melanoma tumor model, NKTR-214 resulted in a CD8^+^ T cell to Foxp3^+^ T_reg_ cell ratio greater than 400, compared to 18 for non-PEGylated IL-2. Furthermore, NKTR-214 provided a 500-fold greater tumor exposure than non-PEGylated IL-2. Combination therapy of NKTR-214 with Nivolumab (an anti-PD-1 antibody) is currently undergoing clinical trials and has demonstrated safety and efficacy in the treatment of solid tumors.^330,331^ In another report, Jerod Ptacin and colleagues utilized an engineered microbial organism with a six-letter semi-synthetic DNA code to create a library of site-specific, click chemistry compatible amino acid substitutions in human IL-2. By covalently modifying IL-2 variants with PEG polymers and screening them, they identified variants with distinct IL-2 receptor specificities and improved pharmacological properties. One notable variant, named THOR-707, exhibits selective engagement of the IL-2Rβ:γc complex without involving the IL-2Rα. When administered in mice, THOR-707 induces significant activation and amplification of CD8^+^ T cells and NK cells, while avoiding the typical T_reg_ cell expansion seen with regular IL-2.^332^ An alternative strategy entails fusing IL-2Rα or antibodies targeting the IL-2Rα binding site of IL-2. In situations where the IL-2Rα binding site is already engaged by the fused IL-2Rα or antibodies, IL-2 will directly bind to the IL-2Rβ:γc complex. Jared Lopes and colleagues developed a novel engineered IL-2 fusion with IL-2Rα, which exhibits potent affinity to the IL-2Rβ:γc complex, while showing no binding to the IL-2Rα:IL-2Rβ:γc complex.^333,334^ This fusion protein, named Nemvaleukin Alfa, underwent clinical trials in combination with Pembrolizumab (an anti-PD-1 receptor antibody) for patients with solid tumors. Nemvaleukin Alfa demonstrated promising efficacy and was generally well tolerated.^335^ Dilara Sahin and colleagues have developed a novel IL-2 antibody fusion protein called NARA1leukin by combining IL-2 with the anti-IL-2 monoclonal antibody NARA1. NARA1leukin completely avoids association with IL-2Rα and displays more potent stimulation of CD8^+^ T cells and NK cells, resulting in robust anti-tumor responses. These highly promising effects have been consistently observed in various pre-clinical cancer models.^336^

Rather than shielding the binding site for IL-2Rα, another approach involves engineering IL-2 with enhanced binding affinity to IL-2Rβ, thereby augmenting its immunostimulatory effects (Fig. 10c). Christopher Garcia’s group employed yeast surface display to identify IL-2 mutants referred to as “super-2”, representing IL-2 superkines characterized by enhanced binding affinity for IL-2Rβ.^251^ Interestingly, the majority of mutations responsible for this increased affinity are located within the core region of the helix bundles rather than at the IL-2:IL-2Rβ interface. Molecular dynamics simulations revealed that the evolved mutations contributed to the stabilization of IL-2 by reducing the flexibility of a helix in the IL-2Rβ binding site, leading to an optimized receptor-binding conformation, closely resembling the conformation observed when IL-2 is bound to IL-2Rα.^251,337^ In a recent study, a purely computational approach was utilized to design IL-2 variants with enhanced affinity for IL-2Rβ, without the need for experimental optimization. Notably, this approach focused on stabilizing the core protein structures rather than the IL-2:IL-2Rβ interface. Through this computational approach, thermostabilized IL-2 variants with up to a 40-fold higher affinity for IL-2Rβ were identified. These IL-2 analogs displayed IL-2Rα-independent activities on T and NK cells both in vitro and in vivo, closely resembling the properties of super-2.^252^ These findings provide additional support for the theory that IL-2Rα enhances the binding affinity of IL-2 to IL-2Rβ by stabilizing an optimized receptor-binding conformation.^251,252^ MDNA11 is a modified version of super-2, where super-2 is fused with albumin to improve its pharmacokinetics and pharmacodynamics properties. MDNA11 displays a reduced or limited stimulation of T_reg_ cells, while inducing a heightened activation of NK cells and naïve CD8 T cells, compared to wild-type IL-2. In pre-established tumor models, MDNA11 effectively controlled tumor growth when administered as a monotherapy or in combination with anti-PD1 or anti-CTLA4 antibodies. The combination therapy exhibited superior efficacy, resulting in sustained tumor clearance through a convenient once-weekly dosing regimen.^337^

Further engineering of super-2 has enabled the development of IL-2 partial agonists, by introducing mutations that weaken the binding to γc, which is useful in adoptive cell therapy (ACT).^338,339^ While IL-2 is known for its ability to stimulate T cell expansion, it can also lead to terminal differentiation, resulting in T cell exhaustion. In ACT, avoiding T cell exhaustion and maintaining a more stem-like state before adoptive transfer is crucial.^340^ The IL-2 partial agonist exhibits a high affinity to IL-2Rβ (determining the EC_50_), but a lower affinity to γc (resulting in reduced E_max_). This property allows it to promote CD8^+^ T cell expansion while preserving a TCF1^+^ stem-like state in vitro, partly by inducing a distinct metabolic profile in these cells compared to wild-type IL-2 (Fig. 10d). Furthermore, when CD8^+^ T cells are cultured with the IL-2 partial agonist ex vivo, their antitumor activity is enhanced following adoptive transfer. These results demonstrate that partial agonists can modify crucial functional properties of pleiotropic cytokines, providing new possibilities for improving ACT outcomes.^256^

In a recent exploration of IL-2 engineering for cancer therapy, Christopher Garcia’s group developed an innovative orthogonal mouse IL-2 cytokine-receptor pair system, by using yeast display to introduce mutations in both the cytokine and the receptor.^341^ These specialized pairs are designed around the principle of receptor-ligand orthogonalization, allowing the receptor to respond to mouse orthogonal IL-2 (ortho-mIL-2), while remaining unresponsive to the wild-type mouse IL-2 (Fig. 10f). Introducing mouse orthogonal IL-2Rβ (ortho-mIL-2Rβ) into T cells facilitated the selective targeting of ortho-mIL-2 to engineer CD4^+^ and CD8^+^ T cells, both in vitro and in vivo, with minimal off-target effects and negligible toxicity.^341^ Subsequently, a human orthogonal IL-2 (ortho-hIL-2) cytokine-receptor (ortho-hIL-2Rβ) pair was developed and tested in CD19-specific chimeric antigen receptor (CAR) T cell therapy (Fig. 10g). The results demonstrated that ortho-hIL-2 led to a dose-dependent increase in the expansion of ortho-hIL-2Rβ^+^ CAR T cells in vivo, reaching up to 1000-fold at two weeks after adoptive transfer into immunodeficient mice with CD19^+^ Nalm6 leukemia xenografts. Notably, ortho-hIL-2 could rescue the anti-leukemic effect even when using an otherwise suboptimal CAR T cell dose. Furthermore, administering ortho-hIL-2 at the time of leukemic relapse following CAR T cell therapy successfully rescued an otherwise failed anti-leukemic response.^342^ A similar approach was employed for the treatment of bulky lymphoma. The outcome revealed that the orthogonal IL-2-IL-2Rβ system led to complete responses in large subcutaneous lymphomas, even with significantly reduced CAR T cell doses, achieved by selectively expanding and activating CAR T cells in vivo.^343^ The unique properties of the orthogonal IL-2-IL-2Rβ system offer a versatile toolbox for exploring the potential of signals from other γc cytokines in ATC, by creating chimeric receptors that retain the extracellular domain of orthogonal IL-2Rβ but replace the intracellular domain with other γc cytokines like IL-4, IL-9, and IL-21 (Fig. 10f).^341^ It was found that the orthogonal chimeric receptor system utilizing IL-9 signals led to the concurrent activation of STAT1, STAT3, and STAT5, assuming characteristics of stem cell memory and T_eff_ cells. Compared to cells with orthogonal IL-2Rβ, cells with the orthogonal chimeric receptor for IL-9 signals demonstrated superior anti-tumor efficacy in two challenging syngeneic mouse solid tumor models of melanoma and pancreatic cancer, even in the absence of lymphodepletion conditioning. These findings underscore the immense potential of the orthogonal cytokine-receptor approach in ATC.^344^

In contrast to cancer treatment, where immunostimulatory effects are desirable, the treatment of autoimmune diseases with IL-2 should be focused on enhancing its immunosuppressive effects, while minimizing its immunostimulatory effects (Fig. 10a). Theoretically, achieving this goal through low doses of IL-2 that activate T_reg_ cells seems plausible. However, in practice, challenges arise due to the narrow therapeutic window. These factors complicate the fine-tuning of IL-2 dosages to strike the right balance between immunosuppression and potential adverse effects.^345–347^ It is hypothesized that increasing the affinity to IL-2Rα or reducing the affinity to IL-2Rβ or γc can effectively enhance T_reg_ cells, while having a lesser effect on T_eff_ cells, thereby achieving the desired immunosuppressive effect (Fig. 10e). Liliane Khoryati and colleagues introduced mutations to IL-2 at the interface with IL-2Rβ, resulting in variants with reduced binding to IL-2Rβ, leading to increased IL-2Rα dependency and enhanced selectivity for T_reg_ cells.^348^ The mutated IL-2 was further fused with Fc to reduce renal clearance. The Fc-fused IL-2 mutant robustly expanded T_reg_ cells in various locations, including the blood, secondary lymphoid tissues, and nonlymphoid autoimmune target tissues in treated mice.^348^ A similar approach was undertaken by Laurence Peterson and colleagues, where reduced affinity to IL-2Rβ was achieved through a single mutation (N88D).^349^ This mutant was further fused with a non-targeted, effector-function-silent human IgG1 and demonstrated promise as a therapy for autoimmune diseases.^349^ In another effort, Christopher Garcia’s group designed a series of IL-2 partial agonists by reducing their affinity to γc.^350^ These IL-2 variants exhibit partial agonism, eliciting a submaximal response at saturating cytokine concentrations. One such IL-2 mutant, IL-2-REH (L18R, Q22E, Q126H), induced the proliferation of Foxp3^+^ T_reg_ cells with reduced activity on NK cells and CD8^+^ T cells (Fig. 10d). This mutant exhibited an increased dependence on IL-2Rα expression and a decreased capacity to overcome negative regulation by SOCS1. The expanded T_reg_ cells retained their suppressive capacity in vitro and demonstrated improved recovery in the dextran sulfate sodium-induced colitis model of IBD.^350^ Bo Zhang and colleagues reported a development of PEGylated IL-2 derivatives with reduced binding to IL-2Rβ.^351^ This modification resulted in enhanced therapeutic efficacy in mouse models of lupus, CIA, and GVHD, without compromising the immune defenses of the host against viral infection.^351^ These diverse approaches aim to fine-tune IL-2 properties to selectively enhance T_reg_ cells function and minimize T_eff_ cell responses, offering potential solutions for achieving the desired immunosuppressive effects in autoimmune disease treatment.

Much like IL-2, IL-15 shares common receptors, IL-2Rβ and γc, to transmit signals from the extracellular to the intracellular environment. Apart from binding to their specific receptors, IL-2Rα and IL-15Rα, the structures of both cytokine receptor complexes bear a striking resemblance. As previously stated, IL-15Rα serves a function similar to that of IL-2Rα by enhancing the binding affinity of IL-15. This similarity has led to the assumption that these cytokines would exhibit comparable functions. In reality, both cytokines indeed stimulate T cell proliferation, promote the development of cytotoxic T lymphocytes, and aid in the maintenance of NK cells. However, they diverge in certain aspects. IL-2 contributes to activation-induced cell death and the fitness upkeep of T_reg_ cells, playing a crucial role in eliminating self-reactive T cells and thereby preventing autoimmune diseases. By contrast, IL-15 plays a critical part in sustaining enduring, high-avidity T cell responses against invading pathogens. This function is achieved by supporting the survival of CD8 memory T cells.^321,352–355^ The disparities between these functions can be attributed to several factors. Firstly, there is a divergence in the distribution of the α receptor subunit. While IL-2Rα is prominently expressed on T_reg_ cells and to a lesser extent on Langerhans cells, endothelial cells, and fibroblasts, IL-15Rα is expressed not only by lymphocytes but also exhibits higher expression on various other cell types, including dendritic cells, monocytes, macrophages, as well as tissue cells like skeletal muscle, lymphoid organ stroma, dermal fibroblasts, nerve cells, and epithelial cells in the lung and intestine. Secondly, IL-2 primarily signals in cis, whereas IL-15 primarily employs an unconventional trans-activation process.^323,353,356^ While both cytokines stimulate diverse lymphocyte and NK subsets, IL-2 primarily influences T_reg_ cell homeostasis and the regulation of T helper cells differentiation. By contrast, IL-15 favors the expansion of CD8 memory cells, T and NK cells.^321,353,355,357^ This functional disparity might be attributed to the differential distribution of the α receptor and their distinct signal behaviors, with IL-2 signaling in a cis manner and IL-15 signaling in a trans manner.^358,359^

In terms of therapeutic development, several cytokine engineering strategies originally applied to IL-2 are also commonly employed for IL-15. For instance, the fusion of IL-15Rα to IL-15 allows the fused protein to activate signaling in IL-2Rβ and γc express cells, bypassing its natural trans-activation mechanism. Erwan Mortier and colleagues designed a potent IL-2Rβ:γc agonist by fusing the Sushi domain of IL-15Rα with IL-15 through a linker. This protein demonstrated some initial antitumor effects, yet its efficacy waned in later stages due to the accumulation of terminally exhausted CD8^+^ T cells marked by high PD-1 expression and Ig mucin-3. However, combining this therapy with a PD-1 antagonist enhanced antitumor activity, leading to clinical trials.^360–362^ In a different approach, rather than fusing the cytokine and receptor, researchers at Hengrui Pharma created a disulfide bond to connect IL-15 with the Sushi domain of IL-15Rα, along with an IgG1 Fc fragment to extend its half-life. This complex displayed favorable pharmacokinetic and pharmacodynamic properties, exhibiting notable antitumor efficacy in both in vitro assessments and in vivo studies.^363^ The high affinity between IL-15 and its α receptor enables the formation of a stable complex even at low concentrations, thus yielding the desired antitumor function even without the need for covalent association.^364^ An alternative approach to fine-tuning IL-15 function involves modifying the affinity between the cytokine and its receptors. For instance, a single mutation, N72D, was discovered to enhance the binding affinity of IL-15 to IL-2Rβ by around 10-fold.^365^ This mutated form was then fused with IL-15Rα and Fc, resulting in the development of ALT-803, which exhibited promising results in preclinical studies involving various tumor models.^366–368^

### IL-10 family cytokines

**Fig. 11:**
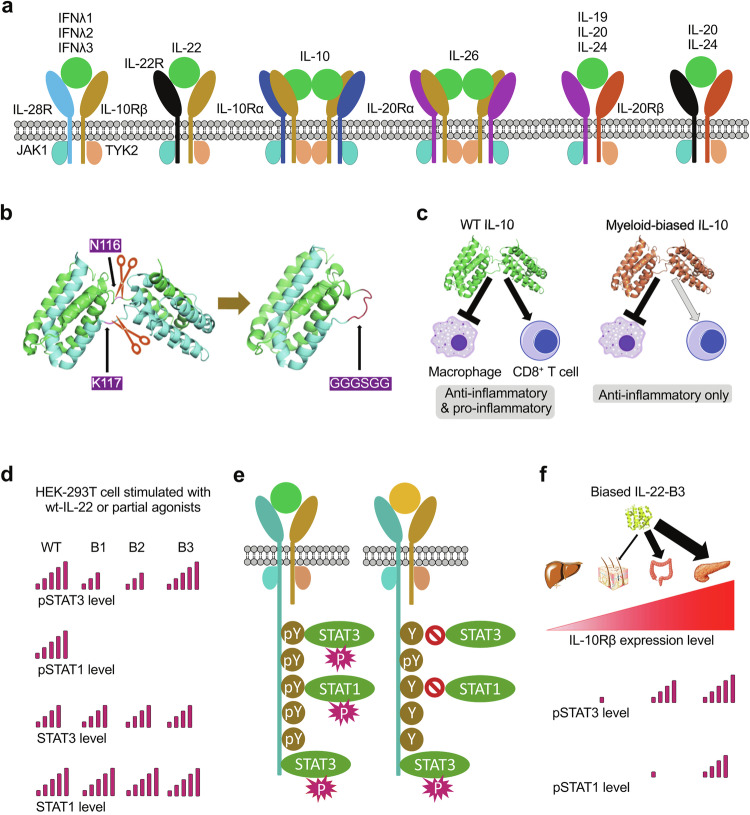
Engineering of IL-10 family cytokines. **a** Receptor binding of IL-10 family cytokines. **b** Conversion of dimeric IL-10 into a functional monomeric IL-10 variant. **c** Decoupling of pro- and anti-inflammatory effects of IL-10 through engineering it (with lower binding affinity to IL-10Rβ compared to wild-type IL-10) with cell type selectivity. This modification results in myeloid-biased activity by suppressing macrophage activation, while avoiding stimulation of inflammatory CD8^+^ T cells. **d** Engineered IL-22 partial agonists (with lower binding affinity to IL-10Rβ compared to wild-type IL-22) exhibit biased signaling, selectively activating STAT3 signals but not STAT1 signals in HEK-293 cells. **e** Mechanism for STAT3-biased functions of IL-22 partial agonists. IL-22 partial agonists induce suboptimal phosphorylation of the intracellular receptor domain, which affects STATs recruitment. STAT3 can bind to the non-phosphorylated receptor through an unconventional mechanism, thus is less affected. **f** IL-22 partial agonists elicit tissue-selective STAT responses in vivo that correlate to the IL-10Rβ expression level in different tissues

The IL-10 cytokine family encompasses IL-10, IL-19, IL-20, IL-22, IL-24, IL-26, and type III IFNs. While these cytokines all involve JAK1 and TYK2 in downstream signaling, they exhibit distinct receptor interactions (Fig. 11a). IL-10 and IL-26 exist as dimers, necessitating the engagement of four receptors to form a functional complex, whereas the remaining members are conventional monomers and require two receptors for their action. While IL-10, IL-22, IL-26, and type III IFNs engage high affinity binding with receptors, specifically IL-10Rα, IL-22R, IL-20Rα, and IL-28R, respectively, they all share a common receptor, IL-10Rβ, albeit with low-affinity binding occurring in the micromolar range.^13,369^ This lower affinity for IL-10Rβ results in the relative instability of the cytokine receptor complex, rendering them unsuitable for cryo-EM samples or obtain high quality crystals. Consequently, despite the structures of these cytokines in complex with high-affinity receptors being reported, there was a notable absence of complete cytokine receptor complexes, inclusive of IL-10Rβ, in the earlier years.^370,371^

The history of engineering IL-10 family cytokines dates back over 20 years when Kristopher Josephson and colleagues transformed dimeric IL-10 into a monomeric protein. This was accomplished by introducing six amino acids (GGGSGG) into the inter-domain linker region of IL-10 between residues N116 and K117 (Fig. 11b).^372^ The engineered IL-10 variant exhibited a similar activity profile to the wild-type cytokine, but it only bound to one molecule of IL-10Rα and one molecule of IL-10Rβ. This monomeric IL-10 variant, which is potentially easier to manipulate compared to the dimeric form, served as a foundational scaffold for generating more potent variants, as demonstrated recently by Claire Gorby and colleagues.^373^ Following the identification of high-affinity variants for the receptor, these variants were subsequently translated back into the dimeric structure resembling the wild-type IL-10. Functional studies showcased a notably enhanced bioactivity profile, especially at lower doses, in monocytes and CD8^+^ T cells when compared to wild-type IL-10. In recent times, Christopher Garcia’s group has employed yeast display techniques to identify cytokine variants of IL-10, IL-22, and IFNλ3 that exhibit high affinity for IL-10Rβ. These cytokine variants displayed notable enhancements in activity. For instance, a specific IFNλ3 variant exhibited up to a 150-fold increase in complex stability, resulting in a remarkable 100-fold improvement in the EC_50_ for pSTAT1 signaling and a 12-fold improvement in the EC_50_ for antiviral activity. Importantly, these cytokine variants serve a pivotal role in stabilizing complete cytokine-receptor complexes, allowing for the preparation of high-quality samples suitable for cryo-EM or crystallography, yielding structures of complete cytokine-receptors complex, which provide valuable insights for further engineering of biased agonists for IL-10, IL-22 and IFNλ3.^256,374,375^

IL-10 exerts both anti-inflammatory and immunostimulatory effects in vivo, due to its capacity to initiate signaling in multiple immune cell types. Its anti-inflammatory effects are primarily attributed to the dampening of monocyte and macrophage activity. Conversely, IL-10 promotes the production of proinflammatory IFNγ by stimulating CD8^+^ T cells.^284,376,377^ Guided by the structure of the IL-10:IL-10Rα:IL-10Rβ complex, a series of mutations were introduced to reduce the cytokine’s affinity for IL-10Rβ, resulting in the creation of biased IL-10 agonists. Notably, the signaling induced by these agonists exhibited a strong correlation with the expression levels of IL-10Rβ on the cell surface. Due to variations in IL-10Rβ expression, certain variants displayed myeloid-biased activity by inhibiting macrophage activation while avoiding the stimulation of inflammatory CD8^+^ T cells (Fig. 11c). This innovative approach effectively uncouples the primary opposing functions of IL-10.^256^

The same approach used for designing biased IL-10 agonists has also proven to be applicable in the development of biased IL-22 agonists.^374^ IL-22 mainly acts within nonhematopoietic cells like epithelial, endothelial, and stromal cells, and is present in various tissues including the pancreas, intestines, lungs, skin, and liver. IL-22 plays a dual role—it protects tissues and can also trigger proinflammatory responses.^377,378^ Based on the crystal structure, a series of IL-22 agonists were generated through systematic mutations of cytokine residues at the IL-22:IL-10Rβ interface to diminish their affinity. The results revealed that the T51A mutation completely abolished the activation of both STAT3 and STAT1, thus designating this variant as an IL-22 antagonist. By contrast, mutations in some other residues were found to weaken, but not entirely abolish, the binding to IL-10Rβ, classifying them as IL-22 partial agonists, which exhibit a notable bias towards STAT3 signaling in comparison to STAT1 (Fig. 11d). The reduced affinity of these partial agonists for IL-10Rβ could potentially lead to a less stable functional cytokine-receptor complex, resulting in a partial decrease in the phosphorylation of JAK1 and TYK2 when compared to the wild-type IL-22. While the decreased phosphorylation of JAK1 and TYK2 could contribute to a less efficient promotion of STAT signaling, it is not the cause of the observed biased signaling phenomenon.^374^ It was discovered that STAT1 phosphorylation is heavily reliant on the phosphorylation of the intracellular domain of receptors, whereas STAT3 can pre-associate with IL-22Rα through a non-canonical mechanism, independent of receptor phosphorylation, by binding to the C-terminal domain of the receptor.^379^ Mutation of all IL-22R cytoplasmic tyrosine residues did not abolish activation of STAT3, in contrast to that of STAT1. Despite triggering robust STAT3 activation, the biased IL-22 variants B1, B2, and B3 all only induced negligible levels of IL-22Rα phosphorylation, compared with wild-type IL-22. This lack of phosphorylation on the intracellular domain of receptors significantly impacts STAT1 phosphorylation, while STAT3 remains less affected (Fig. 11e).^374^ Further investigations revealed that the partial agonist B3 demonstrated in vivo tissue-selective activation of STAT1 and STAT3. This tissue-selective behavior is strongly correlated with IL-10Rβ expression levels, which was highest in the pancreas, followed by the colon, skin, and liver. The variant induced discernibly different STAT1 and STAT3 agonist profiles across tissue types, behaving as a weak STAT3-biased agonist in the pancreas, a strong STAT3-biased agonist in the colon, and a neutral antagonist of STAT1 and STAT3 in the liver (Fig. 11f). This tissue-specific STAT3-biased agonist has the unique ability to uncouple the dual function of IL-22, fostering tissue protection without triggering inflammation. This characteristic holds significant value for therapeutic applications.^374^

### Type I interferons (IFNs)

**Fig. 12:**
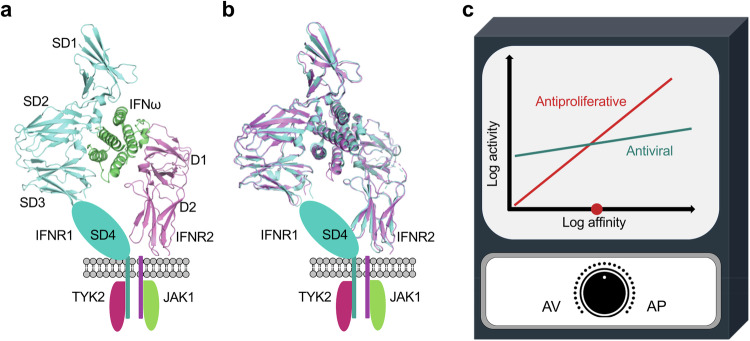
Structure and tunability of type I IFNs. **a** Overall structure of IFNω in complex with IFNAR1 and IFNAR2 (PDB ID: 3SE4). **b** Superposition of IFNω-receptor complex structure (colored in magenta) and IFNα2(YNS) in complex with IFNAR1 and IFNAR2 (colored in cyan, PDB ID: 3SE3). The two structures exhibit remarkable similarity, underscoring a conserved cytokine-receptor recognition mechanism. However, the activities may diverge among different cytokines. **c** Tunability of antiproliferative and antiviral effects of type I IFNs. Both activities are enhanced as cytokine receptor affinity increases, but with a more significant impact on antiproliferative activity

Type I interferons (IFNs) constitute a cytokine family crucial for initiating innate and adaptive immune responses against various pathogens. They also serve as vital regulators in tumor immunity and contribute to the pathophysiology of autoimmune diseases.^380–382^ Type I IFNs have gained approval for the treatment of several human diseases, including viral infections such as HBV and HCV, multiple sclerosis, and certain types of cancer. In the human body, there are 17 different type I IFNs, including IFNα (13 subtypes), IFNβ, IFNω, IFNɛ, and IFNκ.^383–386^ Sequence alignment reveals sequence identities of up to 75% within subtypes but only about 30% between subtypes. Despite this diversity, all type I IFNs share the same receptor pair composed of IFNAR1 and IFNAR2.^383–385^ The extracellular domain of IFNAR1 is distinctive, featuring a unique tandem array of four FnIII subdomains designated SD1 to SD4, while the extracellular domain of IFNAR2 comprises two FnIII domains (D1 and D2) (Fig. 12a). The intracellular domains of IFNAR1 and IFNAR2 associate with TYK2 and JAK1, respectively. Functional studies have illustrated the distinctive roles played by each IFN; they can vary significantly in their potency against different viruses, in their antiproliferative activity, and their ability to activate cells of the immune system.^381,387,388^ However, studies have revealed significant structural similarities among different type I IFNs and their receptor complexes (Fig. 12b).^320,389^ Therefore, the question arises: how do these IFNs manifest distinct functions through the same receptor pair? Studies demonstrated that the varying affinity of diverse IFNs for the IFN receptor subunits is a key factor leading to differences in IFN signaling. This affinity not only determines the receptor’s capacity to capture IFNs at specific concentrations, but also impacts the stability of the functional IFN-receptor complex. This stability could be pivotal for the duration and efficiency of IFN activity.^320,390^ Generally, the binding affinity of IFNs to IFNAR1 is notably lower, residing in the 1–5 millimolar range, except for IFNβ, which boasts an affinity of 50 nM. On the other hand, the binding affinity of IFNs to IFNAR2 ranges from picomolar to nanomolar levels, displaying variations of up to 1000-fold among distinct ligands. The least robust binder to IFNAR2 is IFNα1 (200 nM), while the most potent is IFNβ (0.2 nM). Studies demonstrate a clear linear relationship between binding affinity and antiproliferative activity.^391,392^ Concerning antiviral activity, an increase in binding affinity can slightly enhance potency, but for the majority of IFN types, their affinity has already driven the maximized antiviral effectiveness. In a hepatitis C replication assay, the EC_50_ values exhibited a narrow range of 2- to 6-fold differences among the IFNs (IFNα7: 36 fM, IFNω: 37 fM, IFNα2(YNS, H57Y, E58N, and Q61S): 20 fM, wild-type IFNα2: 116 fM). By contrast, the EC_50_ values for antiproliferative activities differed by more than 1000-fold (IFNα7: 1700 pM, IFNω: 490 pM, IFNα2(YNS): 1.5 pM, wild-type IFNα2: 890 pM). These observations imply that altering the affinity of IFNs for their receptors may yield biased functions.^320,392^

While certain IFNs have gained FDA approval for clinical use, broader adoption has been hindered by side effects and limited efficacy. Nature offers a valuable lesson through its evolution of diverse IFN variants with different functions. Human engineering of these IFNs to possess biased functions holds substantial promise for therapeutic applications. Inspired by nature, there are two potential directions for engineering IFNs (Fig. 12c). The first involves greatly enhancing their affinity to receptors. This enhancement could notably elevate the potency of antiproliferative activities while slightly increasing antiviral activities, albeit potentially reaching a maximum threshold. The second approach is to significantly decrease binding affinity. This might lead to a substantial reduction in antiproliferative activity potency, coupled with only a slight decrease in antiviral effectiveness. The interplay between affinity and function of type I IFNs has been extensively investigated by Gideon Schreiber’s group. By replacing the tail of IFNα2 with that of IFNα8, they achieved a 20-fold increase in affinity for IFNAR2. This modification led to enhanced antiviral and antiproliferative activities.^393^ In another study, the binding region of IFNAR1 for IFNα2 was mapped using alanine scanning. This analysis revealed critical insights into binding affinities, pinpointing specific residues such as H57, E58, and Q61 as enhancers of binding affinity, and F64, N65, T69, and L80 as contributors to decreased binding affinity.^394,395^ Further optimization efforts for IFNα2 resulted in the introduction of mutations (YNS mutant), which conferred a remarkable 60-fold increase in affinity for IFNAR1 without affecting IFNAR2 binding, in comparison to the wild-type IFNα2. The YNS mutant displayed a striking 150-fold rise in antiproliferative potency compared to wild-type IFNα2, but only saw a modest 3.5-fold increase of antiviral activity.^393,396^ Similarly, the mutation K152R in IFNω, which enhances binding to IFNR2, showed remarkedly increase of antiproliferative activity, but only modest increase in antiviral activity.^320^ The mutation R120E had a profound impact, nearly abolishing binding to IFNAR1. This mutation, combined with the replacement of the tail of IFNα2 with that of IFNα8, led to the creation of competitive antagonists for type I IFNs. The antagonist exhibited enhanced affinity for IFNAR2 compared to the wild-type IFN, while showing no detectable binding to IFNAR1. Interestingly, this antagonist maintained the antiviral activity across various cell lines, but did not evoke immunomodulatory or antiproliferative responses, which holds promise as a therapeutic contender for specific viral infections without provoking the immunomodulatory and antiproliferative effects associated with wild-type IFNs.^397,398^

### Engineering cytokines with biased receptor preference

IL-4 plays a crucial role in regulating antibody production, hematopoiesis, inflammation, and the development of T_eff_ cell responses. Its signaling occurs through two distinct heterodimeric receptor complexes: the type I complex composed of IL-4Rα and γc, which activates JAK1 and JAK3, primarily phosphorylating STAT5; and the type II complex, formed by IL-4Rα and IL-13Rα1, which engages JAK1 and TYK2, primarily activating STAT6. IL-4 binds to IL-4Rα with high affinity (K_D_ < 1 nM), whereas the subsequent binding of the IL-4:IL-4Rα complex to either γc or IL-13Rα1 is of relatively low affinity.^399,400^ The type I receptor complex is typically expressed on hematopoietic cells, while the type II complex is predominantly found on non-hematopoietic cells. The type I complex plays a more significant role in regulating T helper 2 (Th2) development, whereas the type II receptor complex is more active in controlling cells responsible for airway hypersensitivity and mucus secretion. Additionally, IL-13 also binds to IL-4Rα and IL-13Rα1 as the type II complex for IL-4 but with a much higher affinity, resulting in functional overlap between IL-4 and IL-13.^400–403^ The differences in receptor distribution on various cells, JAK engagement, and transcription factor activation behavior contribute to the pleiotropy of IL-4, which has limited therapeutic applicability of IL-4.^400,403,404^ Developing IL-4 variants that exclusively induce type I or type II-dependent responses could retain the benefits of IL-4 immunotherapy, while minimizing side effects.

The structures of the IL-4 type I and II complexes, as well as the IL-13:IL-4:IL-13 complex, were solved by Christopher Garcia’s group, providing insights into the molecular mechanisms of cytokine-receptor binding and guidance for cytokine engineering.^400^ Employing a combination of techniques, including a combinatorial library approach and yeast surface display, they identified IL-4 mutants with biased affinity for γc. Sequencing the IL-4-selected variants revealed two unique sequences: the “RQ” and “RGA” variants. RQ-IL-4Rα exhibited a 36-fold higher affinity for γc (K_D_ = 91 nM), and RGA–IL-4Rα had a 3,700-fold higher affinity (K_D_ = 0.89 nM) than IL-4:IL-4Rα. Both RQ and RGA variants displayed significantly reduced binding to IL-13Rα1 (K_D_ = 29,000 nM and 21,000 nM, respectively), suggesting negligible type II receptor binding.^405^ In another design, a 440-fold improvement over wild-type IL-4:IL-4Rα in affinity for IL-13Rα1 (K_D_ = 9.6 nM) but decreased affinity for γc (K_D_ = 6,400 nM) was achieved, demonstrating the successful design of an IL-13Rα1-biased IL-4 variant. Functional studies showed that the enhanced affinity resulted in greater activity than wild-type IL-4. With biased affinities for different receptors, these two types of IL-4 variants exhibit distinct functions, targeting specific cell subsets, potentially enhancing the selectivity of cytokine therapy.^400^ In the JAK-STAT pathway, IL-20 and IL-24 also involve two forms of cytokine-receptor complexes, and the experience gained from studying IL-4 may offer insights for engineering these cytokines.

### Cytokine-based antagonists

The majority of cytokines engage a pair of receptors, typically with one receptor exhibiting high affinity for the cytokine, while the another (typically shared receptor) having lower affinity.^25,245^ One or two mutations at the shared receptor binding site are enough to disrupt the binding of the shared receptor, while maintaining the affinity for the cytokine specific receptor intact. These variants can effectively compete with the natural cytokine by occupying the cytokine specific receptor, yet they fail to form a complete functional cytokine receptor complex, thus acting as antagonists.^406,407^ In the context of therapeutic applications, antibodies have long been used to neutralize signals within the JAK-STAT pathway. In principle, cytokine-based antagonists can serve a similar role to antibodies and be employed for the treatment of certain diseases.^146,147^ The introduction of one or two mutations typically doesn’t induce significant immunogenicity. Therefore, these antagonists can effectively attenuate the excessive signaling of specific cytokines in disease conditions, while maintaining a favorable safety profile. Indeed, a notable example is the development of the IL-4-based antagonist drug known as Pitrakinra, which can be used for the treatment of asthma. The double mutation R121D and Y124D on IL-4 confers its ability to block signaling of both IL-4 and IL-13, by preventing the assembly of IL-4Rα with either γc or IL-13Rα.^406,408^ Other reported cytokine-based antagonists include those for IL-2, IL-15, IL-10, and IFNα2. David Liu and colleagues have created an IL-2 antagonist based on a previous IL-2 mutant with enhanced affinity for IL-2Rα (150-fold lower K_D_ than the wild-type). Additional mutations, V91R and Q126T, with residue substitutions that disrupt the IL-2Rβ and γc binding interfaces, respectively, result in IL-2 antagonists with inhibition constants of approximately 200 nM. These mutants maintain their high-affinity binding to IL-2Rα but do not activate STAT5 phosphorylation, providing a novel approach to T_reg_ cell inhibition.^407^ Suman Mitra and colleagues have worked on developing IL-2 partial agonists by introducing mutations that weaken the binding to γc, resulting in a variant with negligible affinity to γc. This variant functions as an IL-2 antagonist and proves to be more potent than antibodies. It has prolonged survival in a model of GVHD and blocked the spontaneous proliferation of smoldering adult T cell leukemia T cells.^338^ In another effort, Dean Pettit and colleagues demonstrated that mutations D8S and Q108S on IL-15 could individually block the binding of the beta and gamma receptor subunits.^409^ Expanding on this knowledge, a variant of IL-15 (Q101D/Q108D) was identified as an IL-15 antagonist, proving effective in countering CD8^+^ T cell-driven rejection that was resistant to costimulation blockade.^410^

### The coming age of artificial cytokines

The advances in cytokine engineering, which have improved the drug properties of cytokines and reduced the systemic toxicity associated with their pleiotropic nature, have expanded the applications of cytokines in disease treatment. However, there are still challenges in their use for certain disease treatments. The recent technological advancements have opened up new possibilities in the field of artificial cytokines, offering unexplored potential for precise modulation of the JAK-STAT pathway in various diseases.^169,322^ Artificial cytokines offer several advantages over their natural counterparts. First, they can be designed with enhanced drug properties, such as increased stability and improved pharmacokinetics, making them more effective and convenient for therapeutic use. Second, their design can be tailored to have specific affinity for target receptors or cells, enabling more precise and targeted therapeutic interventions. This targeted approach minimizes off-target effects and reduces the toxicity associated with systemic administration, thereby improving the overall safety profile of the treatment. Third, artificial cytokines can be engineered with tunable signaling capabilities, allowing for the desired modulation of cell fates. This flexibility empowers researchers and clinicians to fine-tune the effects of cytokines to achieve the desired therapeutic outcomes. Fourthly, it opens up opportunities to explore non-natural signaling by creating cytokines that interact with non-natural receptor pairs.^411–413^

**Fig. 13:**
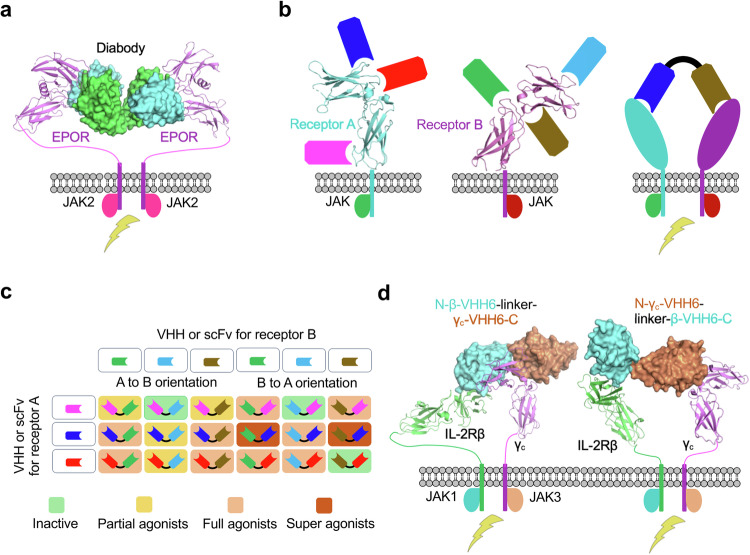
Antibody-based surrogate cytokines. **a** Diabody that binds to EPOR, inducing receptor dimerization and thereby activating the signaling pathway. **b** The principle for surrogate cytokines that induce signals from receptor heteromers. Covalently linking VHH or scFv that bind to distinct receptors can induce receptor dimerization and activate signaling. **c** Combining different VHH or scFv can generate a diverse pool of surrogate cytokines, potentially resulting in varying signal amplitudes within the pathway. **d** By fusing two different VHH or scFv antibodies in tandem, each in different orientations, the geometry of the receptor pair can be altered, leading to distinct signaling effects, exemplified by IL-2 receptors

Cytokines induce receptor dimerization or multimerization, which brings together the intracellular domains, facilitating the formation of the active form of dimeric JAKs; this is the fundamental mechanism for initiating the signaling cascade. The signal behavior can be modified through the engineering of natural cytokines.^169,307,308,319,322^ These observations raise the question of whether synthetic molecules capable of inducing receptor dimerization or multimerization can effectively mimic cytokine’s function. Early evidence addressing this question comes from the study of synthetic EPOR peptide agonists, and antibodies targeting EPOR that can induce signals resembling those of EPO.^414,415^ This concept was further validated by work from Christopher Garcia’s group, in which they extensively investigated the effects of diabodies (covalently linked dimeric antibody VH/VL variable domain fragments) on EPOR signaling (Fig. 13a). Four diabodies induced a range of signal amplitudes, varying from full to minimal agonism, and different extents of STAT5 phosphorylation, STAT5 transcriptional activity, Ba/F3 cell proliferation, and colony formation.^416^ Surprisingly, there was no clear relationship between the signaling amplitude and diabodies’ affinity. Instead, the signaling amplitudes appear to be a result of extracellular receptor dimer proximity (distance) and geometry (orientation) effects. Notably, non-signaling diabodies demonstrated the ability to inhibit the proliferation of erythroid precursors from patients with a myeloproliferative neoplasm due to a constitutively active JAK2 V617F mutant.^416^

One limitation of diabodies is their specificity for cytokine receptor homodimers, whereas in nature, the majority of receptors function as heteromers. A comprehensive study was conducted to create surrogate cytokines for heterodimeric receptors, including receptor complexes for IL-2/IL-15, type I IFNs, and IL-10. This was achieved by tandemly fusing VHH or scFv, which can bind to different receptors, using a short linker (Fig. 13b). The fusion of two binders can be done sequentially in two orders, resulting in a vast pool of surrogate cytokines (Fig. 13c and d).^417^ Out of the 40 surrogate cytokines tested for IL-2 receptors, 28 demonstrated agonistic properties, resulting in an impressive ~70% hit rate. These agonists spanned a range of activities, from minimally active (3 ligands) to approximately half of the E_max_ relative to IL-2 (5 ligands), full E_max_ (17 ligands), and even supraphysiological E_max_ (3 ligands).^417^ On the other hand, the success rate for surrogate cytokines targeting type I interferons was comparatively lower. However, some of these surrogate cytokines displayed excellent antiviral activity against SARS-CoV-2, while exhibiting restrained anti-proliferative and pro-inflammatory effects.^417^ The modular platform employed in the study also allowed for the engineering of surrogate ligands, compelling the assembly of an IL-2Rβ:IL-10Rβ heterodimer, which does not naturally occur. This engineered receptor complex effectively signaled through pSTAT5 on T and NK cells. Similar to diabodies, the E_max_ of these surrogate cytokines was influenced by the geometry of the signaling complex, but no correlation was observed between affinity and E_max_.^416,417^ The successful development of these surrogate agonists provides theoretical basis for the use of synthetic molecules as cytokines, opening up unexplored avenues in this field. These surrogate agonists have unveiled a remarkably broad signaling plasticity and downstream functional diversification that may hold therapeutic potential.^417^

One notable aspect is that cytokines in the JAK-STAT pathway are primarily helical proteins. Normally helical proteins are tightly packed and tend to exhibit greater stability compared to proteins with more disordered secondary structures such as loops and turns. It seems that helical proteins tend to have higher success rates in protein scaffold design, protein binder design, or protein modification, such as grafting with residues, motif or protein domains from another protein.^418–421^ Protein design techniques have the potential to revolutionize the development of artificial cytokines. The Rosetta software suite, developed by David Baker’s group, offers a robust platform with diverse applications, encompassing the design of protein scaffolds, therapeutic protein binders, hyperstable proteins, and enzymes.^418,422^ Notably, protein binder design is closely linked to artificial cytokine design, as cytokines essentially act as protein binders that interact with receptor pairs. Pioneering work in protein binder design achieved remarkable success by creating small protein binders for the stem of influenza virus hemagglutinin (HA) in the low-nanomolar range.^419,423^ Over a decade, binder design has significantly matured and become more robust, benefiting from various factors, including advanced docking methods like RIFDock, improved energy functions within the software, advancements in DNA synthesis techniques, and a readily accessible mini protein library serving as ideal scaffolds for binders.^420,421,424–428^ In recent times, AI approaches for protein structure prediction and design may hold promise for cytokine design.^429–433^ Notably, methods that ‘hallucinate’ proteins using trRosetta, RoseTTAFold or RFdiffusion have demonstrated particular usefulness for designing proteins with novel configurations, and might be valuable for scaffold building, loop building or helix connections during cytokine design.^431–433^ More recently, a graph neural network-based protein design method called ProteinMPNN (message-passing neural network), developed by David Baker’s group, has demonstrated significant efficacy in rapidly designing protein sequences for a given structure. Experimental test results show soluble protein expression for many sequences generated by ProteinMPNN, ranging from small proteins to large protein complexes.^434,435^ This method could serve as an ideal companion to the hallucination approach for artificial cytokine design. The combination of these cutting-edge techniques, which has demonstrated remarkable success in designing protein binders, holds great promise in unlocking a new realm of possibilities for the creation of novel cytokines with potential applications in therapy and beyond.^436^

**Fig. 14:**
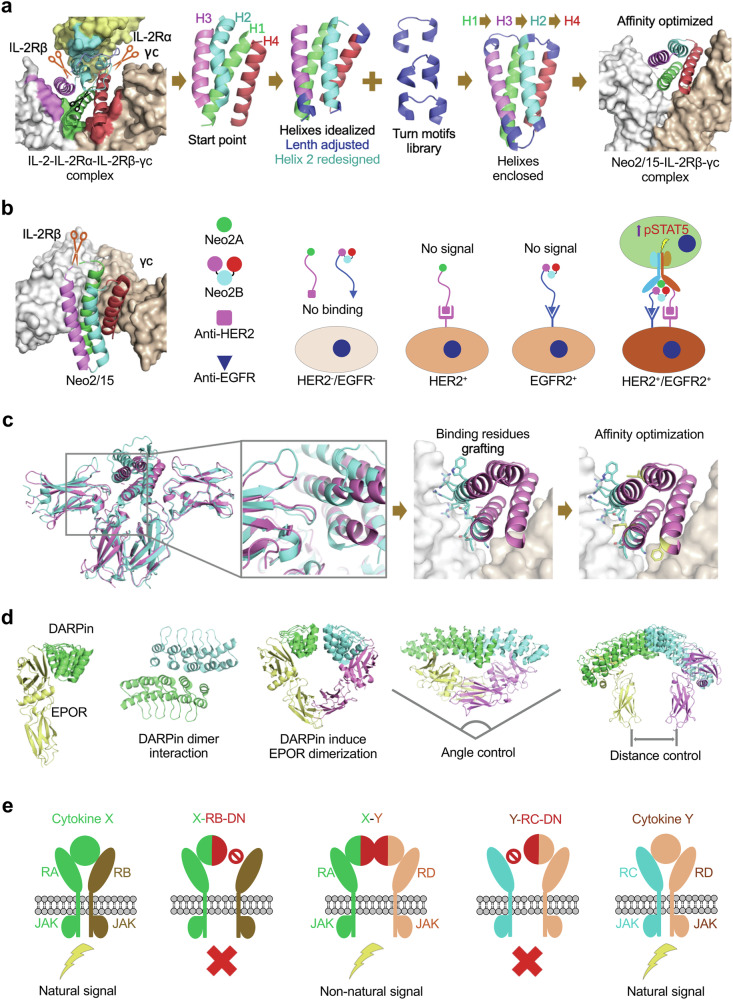
Design and engineering of artificial cytokines. **a** Design of Neo-2/15. The design begins with four helices from IL-2 as a starting point, with the loops removed to facilitate the reorganization of these helices. The helices are optimized, with a specific focus on redesigning helix 2 and idealizing the others. Short loops and helix segments enclose the helices to create early IL-2/15 mimics. The resulting cytokine mimics, composed of four helix bundles, are further optimized to enhance stability and affinity, ultimately leading to the development of Neo-2/15. **b** Development of a conditionally active IL-2/15 agonist. Neo-2/15 is divided into two segments, named Neo2A and Neo2B, both of which are inactive when separate. However, when brought together, they form a four-helix complex and become an active IL-2/15 agonist. When fused with target protein binders, such as nanobodies that target tumor antigens, they can translocate to the site where the antigen is presented, forming a functional IL-2/15 agonist complex that stimulates local immune activity. **c** Engineering an IL-4 agonist via Neo2/15 modification. Residues corresponding to those binding to IL-4Rα (shown in cyan) were grafted onto Neo-2/15, yielding an IL-4 agonist. Subsequent optimization efforts resulted in a high-affinity IL-4 agonist (new mutations are shown in yellow). **d** Design of DARPin-based surrogate cytokines that can control the angle and distance between EPOR pairs. **e** Activating non-natural signaling through non-natural receptor composition by fusion of two dominant negative (DN) cytokines

A notable breakthrough example of such work is the de novo design of IL-2/IL-15 mimics, pioneered by the collaborative efforts of Christopher Garcia’s group and David Baker’s group.^411^ As previously discussed, both IL-2 and IL-15 exhibit two binding forms: one involving their private α receptor, IL-2Rβ, and γc, and the other consisting of IL-2Rβ and γc only. In cancer therapy, prioritizing the second form and avoiding the first form is crucial, to decouple pleiotropic effects, thereby enhancing efficacy and reducing toxicity.^25,245,251^ Native IL-2 consists of four helices connected by loops, forming a N-terminal helix (H1), long loop, helix (H2), short loop, helix 3 (H3), long loop, and C-terminal helix (H4) architecture. The IL-2Rα interacts with a surface formed by H2 and two long loops, IL-2Rβ interacts with H1 and H3, and γc interacts with H1 and H4 (Fig. 14a).^245^ A possibility arises to redesign IL-2 by removing these long loops, which may result in a safer cytokine mimic that does not bind to IL-2Rα. Using all helices as starting points, the design process entailed using Rosetta to idealize H1, H3, and H4, while remodeling the bent H2 with a straighter and more optimal helix.^411^ Key residues interacting with IL-2Rβ and γc were retained as hot spot residues, and short loops with or without helix elements at the terminals were used to enclose two helices, resulting in dozens of 4-helix bundle variants capable of binding with IL-2Rβ and γc chimeric receptors at low-nanomolar concentrations.^411^ After optimization to increase binding affinity and repacking the core of helix bundles for enhanced protein stability, higher-affinity hyper-stable variants of the de novo mimics were obtained, exhibiting distinct topology and minimal sequence identity to human or mouse IL-2 (Fig. 14a). One noteworthy cytokine mimic, Neo-2/15, demonstrated high affinity binding to human and mouse IL-2Rβ:γc (with K_D_ ≈ 19 nM and K_D_ ≈ 38 nM, respectively), surpassing the corresponding native IL-2 cytokines, while not interacting with IL-2Rα. Neo-2/15 demonstrated superior therapeutic efficacy to IL-2 in mouse models of melanoma and colon cancer, exhibiting reduced toxicity and minimal immunogenicity, while also showing robust antitumor activity in both monotherapy and combination with PD-1 blockade in clinical trials.^411,437^

Recently, an innovative Neo-2/15 based conditionally active IL-2 mimic was developed by David Baker’s group.^438^ The Neo-2/15 is split into two fragments: Neo2A, containing H1, and Neo2B, containing H3-H2-H4. Independently, neither fragment shows any activity even at high concentrations. However, when IL-2Rβ and γc are present, the two components combine to form a fully active IL-2 mimic (Fig. 14b). A conditionally active IL-2 mimic can be achieved by fusing Neo2A and Neo2B with tumor-associated antigen-binding proteins, such as nanobodies. For example, by fusing Neo2A with a HER2 binding protein and Neo2B with an epidermal growth factor receptor (EGFR) binding protein, this design functions like an ‘AND’ logic-gate, as the IL-2 mimic is activated only when both tumor-associated antigens are present within the cells or neighboring cells in the TME, bringing the two components close together (Fig. 14b). This conditional activity allows for precise targeting and concentration in tumor cells, minimizing side effects caused by systemic drug distribution. Animal models have demonstrated the impressive anti-tumor effects of the IL-2 split mimic, potentially revolutionizing cancer therapy in the future.^438^

The success of Neo-2/15 design further led to the development of a biased IL-4 mimic with exclusive binding to the type I receptor complex (Fig. 14c).^439^ IL-4 signals through two receptor complexes: the type I complex composed of IL-4Rα and γc, and the type II complex composed of IL-4Rα and IL-13Rα1 chains.^400–402^ By designing an IL-4 mimic that engages only with the type I form, the pleiotropic effects of IL-4 can be decoupled, potentially benefiting therapeutic purposes. The initial design involved grafting 14 residues implicated in the IL-4:IL-4Rα interface onto Neo-2/15, while preserving residues implicated in the Neo-2/15:γc interface. Subsequent yeast surface display selection resulted in 16 mutations being introduced into Neo-2/15, to create the IL-4 mimic. Compared to native IL-4, the IL-4 mimic exhibits 121-fold weaker binding affinity to IL-4Rα (K_D_ = 58 nM) but comparable affinity (K_D_ = 80 nM) for the IL-4Rα:γc complex. The IL-4 mimic exclusively signals through the type I IL-4 receptor complex, providing new insights into differential IL-4 signaling through type I versus type II receptors. Additionally, the hyperstable properties of IL-4 mimic make it suitable for incorporation into sophisticated biomaterials that require heat processing, such as 3D-printed scaffolds.^439^

Despite the natural cytokines in the JAK-STAT pathway comprising only four or five helices, artificial cytokines can be designed with larger size, increased protein surface area, and variations in their structure, such as homodimers, heterodimers, repeated motifs, or altered shapes. This allows for the regulation of distance, geometry, or composition within the cytokine-receptor interaction, influencing downstream signaling. A pioneering work in this exploration was conducted by a collaboration between groups from David Baker and Christopher Garcia, where they designed the ankyrin repeat protein (DARPin) scaffold as a surrogate cytokine, capable of systematically controlling the orientation and the distance of two EPORs (Fig. 14d).^440^ Ankyrin repeat proteins are built from tightly packed repeats of 33 amino acid residues, comprising a structural unit consisting of a β-turn followed by two antiparallel α-helices, making them suitable for designing protein binders.^441^ Using yeast-display DARPin libraries, low-affinity EPOR binders were identified and further optimized to high-affinity EPOR binders through random mutagenesis and DNA-shuffling techniques. The resulting DARPin variant was used to develop EPOR agonists, either by designing DARPin-based binder dimers or a single large-size DARPin containing two EPOR binding sites. Although the DARPin’s binding to EPOR differs from the natural ligand of EPO, it still activates EPOR and induces downstream signaling. Given that DARPin is a repeat modular protein and capable of self-assembling oligomers, DARPin-based EPOR agonists can be designed in various sizes and geometries by controlling the number of repeat units and dimer interactions (Fig. 14d). Consequently, a broad range of full, partial, biased, and stage-selective agonists was developed, facilitating a comprehensive study of ligand-receptor spatial relationships and signaling.^440^

The human genome encodes approximately forty different JAK-STAT pathway cytokine receptors, theoretically giving rise to around 800 unique homo- and hetero-dimeric cytokine receptor pairs that could signal through different JAK/STAT combinations. However, in reality, there are only about 40 cytokine receptor pairs for approximately 50 natural ligands.^13,25^ This leaves a vast area of unexplored territory, as we are still uncertain about the signals induced by non-natural receptor pairs and their consequences on gene expression and cell fate. Artificial cytokines present an exciting opportunity for such exploration.^412^ As previously mentioned, Christopher Garcia’s group has employed antibody-based techniques to develop surrogate cytokines, which can bind to non-natural receptor pairs, enabling the induction of specific signals.^401^ They have also designed cytokine based artificial cytokines with the ability to bind to non-natural receptor pairs (Fig. 14e).^412^ To achieve this, they introduced mutations to abolish the IL-13Rα1 and γc binding sites of IL-4, γc binding sites of IL-2, and IFNAR1 binding sites of IFN. But they ensured the preservation of IL-4Rα for IL-4, IL-2Rβ for IL-2, and IFNAR2 for IFN. As a result, these engineered cytokines lack signaling activity on their own, leading to what they refer to as dominant negative (DN) cytokines. By fusing two such DN cytokines with short linkers, they facilitated the dimerization of the non-natural receptor pairs, resulting in a distinct signaling response profile compared to natural cytokines (Fig. 14e).^412^ The ability to engineer cytokines with tailored signaling properties holds great potential for advancing our understanding of immune responses and developing innovative therapeutic interventions.

## Conclusions and future perspectives

Over the past three decades, our understanding of the JAK-STAT pathway has expanded significantly in various aspects. We’ve made substantial strides in comprehending the functions and their relationships to human diseases, such as autoimmune diseases, hematological disorders, and immunological responses against tumors. These insights have enabled us to delve into the dysregulation of the pathway caused by factors like gene mutations and its impact on the pathological characteristics. The ongoing advancements in the structural biology of the JAK-STAT pathway provide a comprehensive depiction of the intricate processes. The elucidation of the structure of key proteins within this pathway has allowed us to gain a deeper comprehension of cytokine-receptor recognition, signal transduction mechanisms, and the structural basis for pathway activation and inhibition, providing a foundation for the development of precise and effective therapeutic strategies. Coupled with advancements in protein engineering techniques, the rise of cytokine engineering is poised to transform the pharmaceutical landscape, making formerly unusable cytokines viable for therapeutic applications that are now undergoing clinical trials.

Despite these strides, several crucial aspects warrant further exploration. First, elucidating the structural intricacies of many cytokine-receptor interactions remains a paramount challenge. This is a critical step in unraveling the nuanced ways in which these signaling molecules engage with their receptors to initiate downstream cellular responses. Second, the dynamic nature of the JAK-STAT pathway activation and the regulatory mechanisms governing these pivotal signaling molecules are areas for exploration. Next, the intricate details of STAT-DNA recognition, particularly in scenarios where STAT heterodimers interact with DNA, represent another significant area requiring focused investigation. This is crucial for deciphering how these transcription factors contribute to the regulation of gene expression, influencing cellular functions and fate. Recent advancements in structural biology, such as cryo-EM, have played a crucial role in elucidating the structures of the JAK-STAT pathway proteins, such as the full-length of mJAK1, IL-6, and IL-10 cytokine-receptor complexes.^24,220,256^ Unlike traditional macromolecular structure determination methods, cryo-EM does not require crystals and only requires small sample amounts. However, the overall resolution of cryo-EM structures may lag behind that of crystall structures. Recently developed AI-based structure prediction methods, such as AlphaFold 2 and RoseTTAFold, have demonstrated remarkable performance in predicting protein structures, particularly for individual protein domains.^426,430^ These protein prediction methods can be seamlessly integrated with cryo-EM data, where cryo-EM provides the overall structure, and predicted protein structures fill in the details. This combined approach holds promise for elucidating the molecular mechanisms of the JAK-STAT pathway. The recent releases of RoseTTAFold all-atom and AlphaFold 3 may present new opportunities for understanding the molecular mechanisms of the JAK-STAT pathway.^442,443^ These updated AI-based structure prediction tools are not limited to predicting protein structures but can also predict structures of protein-small molecule complexes and protein-nucleic acid complexes. This capability may prove invaluable in research on STAT-DNA recognition.

In terms of therapeutic development, the overarching objective in the development of cytokine and cytokine mimic-based therapeutics remains the reduction of toxicity, by decoupling pleiotropy and enhancing pharmacological properties to increase efficiency. Techniques such as rational mutagenesis, yeast display, PEGylation, immunocytokines, Rosetta modeling, AI-based protein structure prediction and design have played pivotal roles in modifying cytokines or creating novel cytokine agonists to achieve these objectives. An important observation from past cytokine engineering endeavors is the significant impact of cytokine-receptor affinity on signaling. Cytokine-receptor affinity undeniably influences receptor engagement choices. Cytokines such as IL-2, IL-4, IL-20, and IL-24 can engage with more than one receptor complexes. Modifying a cytokine’s affinity for specific receptors can result in cytokine agonists with a specific preference for certain receptor pairs, which is particularly crucial for decoupling pleiotropy. Moreover, even for cytokines that interact with a single receptor pair, cytokine-receptor affinity also greatly influences downstream signaling. By manipulating cytokine-receptor affinity, it is possible to develop various cytokine agonists, including super agonists, full agonists, partial agonists, and biased agonists, each exerting different therapeutic effects.^168^ Yet, the relationship between affinity and downstream signaling for most cytokines remains unclear, except for certain cytokines such as type I IFNs. Clarifying this relationship and its underlying molecular mechanisms represents an area for research, potentially leading to the development of improved cytokine agonists for therapeutic use. Innovative approaches, including orthogonal cytokine-receptor pairs, conditionally active agonists and artificial cytokine-based therapeutics may emerge as an unexplored wealth of possibilities, destined to reveal its true potential in the future. Additionally, exploring cytokine agonists that bind and induce signals for non-natural receptor pairs presents another intriguing avenue for investigation. The therapeutic potential of such non-natural signals warrants further exploration.
