# Correction: The JAK-STAT pathway: from structural biology to cytokine engineering

**DOI:** 10.1038/s41392-024-01975-1

**Published:** 2024-10-17

**Authors:** You Lv, Jianxun Qi, Jeffrey J. Babon, Longxing Cao, Guohuang Fan, Jiajia Lang, Jin Zhang, Pengbing Mi, Bostjan Kobe, Faming Wang

**Affiliations:** 1 Center for Molecular Biosciences and Non-communicable Diseases Research, Xi’an University of Science and Technology, Xi’an, 710054 Shaanxi China; 2 Xi’an Amazinggene Co., Ltd, Xi’an, 710026 Shaanxi China; 3 CAS Key Laboratory of Pathogen Microbiology and Immunology, Institute of Microbiology, Chinese Academy of Sciences, 100080 Beijing, China; 4 The Walter and Eliza Hall Institute of Medical Research, Parkville, VIC 3052 Australia; 5 School of Life Sciences, Westlake University, Hangzhou, 310024 Zhejiang China; 6 Immunophage Biotech Co., Ltd, No. 10 Lv Zhou Huan Road, Shanghai, 201112 China; 7 School of Pharmaceutical Science, Hengyang Medical School, University of South China, Hengyang, 421001 Hunan China; 8 School of Chemistry and Molecular Biosciences, Institute for Molecular Bioscience and Australian Infectious Diseases Research Centre, University of Queensland, Brisbane, QLD 4072 Australia

**Keywords:** Molecular biology, Structural biology

**Correction to:** *Signal Transduction and Targeted Therapy* 10.1038/s41392-024-01934-w, published online 21 August 2024

After online publication of the Article,^1^ the authors received feedback from a reader that an inadvertent error was found on earlier understanding on the organizing principle of common beta cytokine signaling. A recent study published in the journal Molecular Cell updates our understanding of the hexameric structural model of the βc family cytokine-receptor complex signaling transduction.^2^ Unfortunately, we did not catch these new findings during the revision of our manuscript. Here, we update Fig. 4 and its legend of the original Article and correct the related descriptions of the βc family cytokine signaling model in the main text. These corrections did not affect any of our key conclusions presented in the original Article. We apologize for any inconvenience this has caused. The original Article has been corrected.

1. The inaccurate descriptive sentence in the second paragraph (the right column) on Page 7:
  However, certain receptors, such as IL-2Rα, IL-3Rα, IL-5Rα, IL-6Rα, IL-11Rα and IL-15Rα, lack the necessary regions for direct binding of JAKs or STATs.
  The accurate descriptive sentence is as below:
  However, certain receptors, such as IL-2Rα, IL-6Rα, IL-11Rα and IL-15Rα, lack the necessary regions for direct binding of JAKs or STATs.
2. The inaccurate description of the whole third paragraph (the right column) on Page 8:
  As GM-CSFRα does not involve JAKs binding or STATs recruitment, questions arise regarding how the cytokine-receptor complex transmits information.234-236 First, the C-termini of βc extracellular domains are approximately ~120 Å apart, a distance that appears too far to allow the formation of the JAK dimer necessary for signaling. Second, if the ~120 Å distance is deemed sufficient for signal transduction, the dimeric βc molecule already exhibits this distance at its D4 for signaling; thus, why does it necessitate GM-CSF and GM-CSFRα to form a hexamer? Moreover, how the signaling is initiated upon the ligand binding and how the signaling is subsequently turned off? Consequently, it appears that the kinase activity is not exerted by two distant JAKs binding to the intracellular domain within the same dimeric βc.234 Moreover, no other JAK pair with a closer distance than ~120 Å was observed in the GM-CSF-receptor hexamer complex. This observation raises doubts about the hexameric GM-CSF-receptor complex being the signaling complex for GM-CSF.234 However, higher-order oligomeric states (dodecamers) were observed in the crystal lattice, featuring contacts between GM-CSF and GM-CSF, as well as GM-CSFRα and GM-CSFRα, bringing two D4 of βc close together (Fig. 4c). The proximity between the two D4 domains of βc from two GM-CSF-receptor hexamers is crucial for maintaining the close distance of JAK molecules, a critical factor for JAK activation. It is suggested that the dodecamer is the minimal unit for GM-CSF signaling, and even higher oligomeric states may exist for GM-CSF signaling. The formation of the cytokine-receptor hexamer is a prerequisite for the formation of signaling complexes.234,237
  The accurate description of the whole third paragraph (the right column) on Page 8 is as below:
  For two decades, it was wrongly assumed that the specific receptors of βc family cytokines (IL-3Rα, IL-5Rα, and GM-CSFRα) did not link with JAKs and that signaling of these cytokines depended only on the shared βc.234-236 However, in the dimeric βc structure, the intracellular domains are approximately 120 Å apart, which is too far to enable signaling (Fig. 4a). Higher-order oligomeric states (dodecamer) were identified in the crystal lattice, potentially bringing the two βc intracellular domains closer together. Therefore, the dodecamer was proposed as the smallest unit responsible for GM-CSF signaling.234-237 This incorrect notion persisted until Christopher Garcia's group published a paper on the organizing principle of common beta cytokine signaling.238 The authors used cryo-EM to establish the structures of the βc family cytokine-receptor complex at 3.6–3.8 Å resolutions.238 The cytokine-receptor complex is a hexameric structure with two copies of the cytokine, receptor, and βc molecules, comparable to the crystal structure. Cryo-EM did not reveal increased oligomeric states, which were thought to bring the two βc intracellular domains closer together for signaling. Thus, the dodecameric cytokine-receptor structure is most likely due to crystal constraints and does not present in physiological settings. This conclusion was reinforced by single-molecule imaging experiments.238 Live-cell micropatterning tests were used to determine if the specific receptors could connect with JAKs, and the results revealed that all three unique receptors could bind JAKs significantly above baseline. The role of particular receptor intracellular regions in signaling was verified by connecting the transmembrane region and intracellular domains of βc family cytokine receptors to the extracellular domain of the orthogonal IL-2 receptor system.238 When orthogonal IL-2 was added, dose-dependent signaling occurred. The βc family cytokine-receptor complex, with its hexameric shape, is sufficient for cytokine signaling (Fig. 4c), indicating that particular receptors interact with JAKs and contribute to signaling.
3. Incorrect image of Fig. 4:

Fig. 4 Structure of the cytokine-receptor complex of βc cytokines exemplified by the GM-CSF-receptor complex (PDB ID: 4NKQ). Domain 1 (D1, orange) of GM-CSFRα is modeled using D1 of GM-CSFRα from the GM-CSF:GM-CSFRα complex (PDB ID: 4RS1). a Hexamer of the GM-CSF-receptor complex from a side view. The CHR modules of GM-CSFRα (D2 and D3, orange) interact with GM-CSF (colored in magenta) at site 1, while the CHR modules of βc (D4 from itself and D1 from the partner βc) interact with GM-CSF at site 2. Each βc dimer is bound by two molecules of JAK2, but two JAK2 molecules are distant from each other, as βc D4 domains are approximately 120 Å apart. b Cytokine-receptor hexamer of the GM-CSF-receptor complex from a top view. The CHR modules of βc are bridged by the D2 and D3 domains of βc. c The dodecameric GM-CSF-receptor assembly is observed in the crystal lattice. In this arrangement, D4 of βc from two hexamers is in close proximity. This close juxtaposition is suggested to be crucial for maintaining the necessary proximity for JAK pairs, a key factor in signaling. Hence, the dodecamer is suggested as the minimal unit for GM-CSF signaling
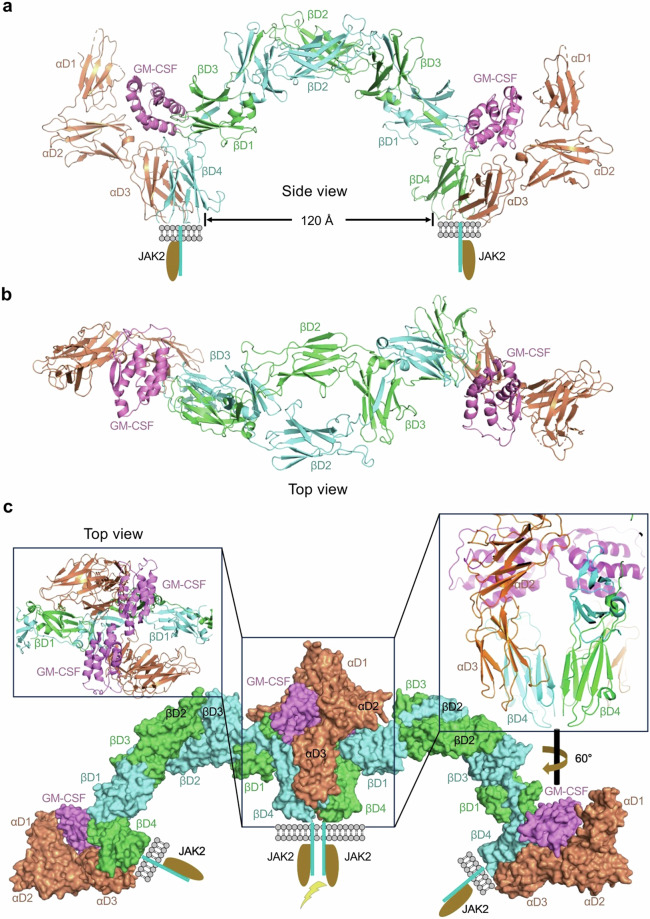


Correct image of Fig. 4 is as below:
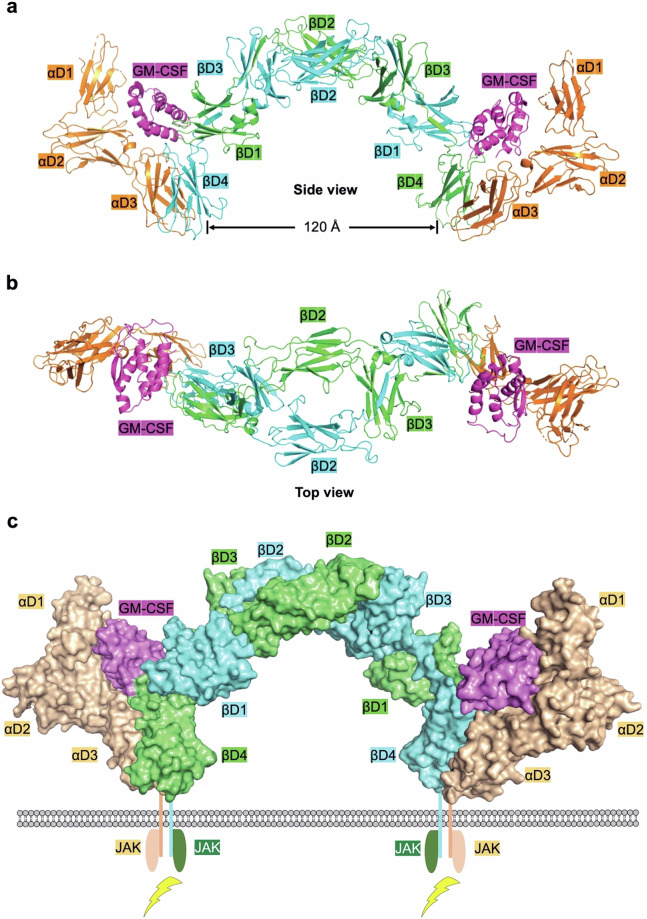


Fig. 4 Structure of the cytokine-receptor complex of βc cytokines exemplified by the GM-CSF-receptor complex (PDB ID: 4NKQ). Domain 1 (D1, orange) of GM-CSFRα is modeled using D1 of GM-CSFRα from the GM-CSF:GM-CSFRα complex (PDB ID: 4RS1). a. Hexamer of the GM-CSF-receptor complex from a side view. The CHR modules of GM-CSFRα (D2 and D3, orange) interact with GM-CSF (colored in magenta) at site 1, while the CHR modules of βc (D4 from itself and D1 from the partner βc) interact with GM-CSF at site 2. Each βc dimer is bound by two molecules of JAK2, but two JAK2 molecules are distant from each other, as βc D4 domains are approximately 120 Å apart. b. Cytokine-receptor hexamer of the GM-CSF-receptor complex from a top view. The CHR modules of βc are bridged by the D2 and D3 domains of βc. c. Signaling complex for βc cytokines exemplified by the GM-CSF-receptor complex. The intracellular domains of both GM-CSFRα and βc associate with JAKs, which is critical for signaling
